# Förster Resonance Energy Transfer-Based Single-Cell Imaging Reveals Piezo1-Induced Ca^2+^ Flux Mediates Membrane Ruffling and Cell Survival

**DOI:** 10.3389/fcell.2022.865056

**Published:** 2022-05-13

**Authors:** Heon-Su Kim, Jung-Soo Suh, Yoon-Kwan Jang, Sang-Hyun Ahn, Gyu-Ho Choi, Jin-Young Yang, Gah-Hyun Lim, Youngmi Jung, Jie Jiang, Jie Sun, Myungeun Suk, Yingxiao Wang, Tae-Jin Kim

**Affiliations:** ^1^ Department of Integrated Biological Science, Pusan National University, Pusan, South Korea; ^2^ Institute of Systems Biology, Pusan National University, Pusan, South Korea; ^3^ Department of Cell Biology, School of Medicine, Zhejiang University, Hangzhou, China; ^4^ Department of Mechanical Engineering, Dong-Eui University, Pusan, South Korea; ^5^ Department of Bioengineering, Institute of Engineering in Medicine, University of California, San Diego, La Jolla, CA, United States; ^6^ Department of Biological Sciences, Pusan National University, Pusan, South Korea

**Keywords:** FRET, biosensors, Piezo1, calcium, membrane ruffling

## Abstract

A mechanosensitive ion channel, Piezo1 induces non-selective cation flux in response to various mechanical stresses. However, the biological interpretation and underlying mechanisms of cells resulting from Piezo1 activation remain elusive. This study elucidates Piezo1-mediated Ca^2+^ influx driven by channel activation and cellular behavior using novel Förster Resonance Energy Transfer (FRET)-based biosensors and single-cell imaging analysis. Results reveal that extracellular Ca^2+^ influx *via* Piezo1 requires intact caveolin, cholesterol, and cytoskeletal support. Increased cytoplasmic Ca^2+^ levels enhance PKA, ERK, Rac1, and ROCK activity, which have the potential to promote cancer cell survival and migration. Furthermore, we demonstrate that Piezo1-mediated Ca^2+^ influx upregulates membrane ruffling, a characteristic feature of cancer cell metastasis, using spatiotemporal image correlation spectroscopy. Thus, our findings provide new insights into the function of Piezo1, suggesting that Piezo1 plays a significant role in the behavior of cancer cells.

## Introduction

Piezo1, a mechanosensitive channel, has been continuously investigated since its first cloning in 2010 (Coste et al., 2010). Piezo1 translates mechanical stimulation from the peripheral microenvironment into an electrical signal, and is a non-selective cation channel that is permeable to Na^+^, K^+^, Mg^2+^, and Ca^2+^, with a slight preference for Ca^2+^(Coste et al., 2010; Coste et al., 2012; Lin et al., 2019). Mechanosensitivity of the Piezo1 channel is involved in various physiological functions, such as red blood cell volume, epithelial homeostasis, blood pressure regulation, vascular and lymphatic development, and bone formation (Ranade et al., 2014; Cahalan et al., 2015; Wang et al., 2016; Gudipaty et al., 2017; Nonomura et al., 2018; Sun et al., 2019). Impairment of Piezo1 activity resulting from inherited mutations and genetic manipulations has been associated with hereditary xerocytosis, lymphatic dysplasia, and hemolytic anemia (Zarychanski et al., 2012; Andolfo et al., 2013; Lukacs et al., 2015; Alper, 2017).

Two models have been suggested to understand the gating mechanism by which Piezo1 opens a central pore in response to mechanical force. The first model is the force-from-lipid model (Kung et al., 2010; Anishkin et al., 2014), where the tension of the cell membrane changes and the membrane flattens and contracts due to external stimuli; the interaction between 24 transmembrane helices consisting of the arm of Piezo1 and membrane lipids is altered and the arm is transformed into a flat shape. Eventually, the dome-shaped Piezo1 flattens, fulfilling the free energy required for channel gating and leading to the passage of ions (Lewis and Grandl, 2015; Cox et al., 2017; Lin et al., 2019). In other words, the channel reacts to a very local stimulus, such as a change in membrane tension, and carries out mechanotransduction (Cox et al., 2016; Syeda et al., 2016). The second model is the force-from-filament model, which suggests that Piezo1 physically binds to ambient non-compliant structures such as the extracellular matrix and cytoskeleton structures, and mechanogating occurs in response to mechanical stimuli from the structures or movement of the membrane-bound channels relative to the stationary structures (Markin and Hudspeth, 1995; Gillespie and Walker, 2001; Liang et al., 2013; Jin et al., 2017). In a study by Wang et al. (2020) endogenous Piezo1 was found to be bound to a mechanotransduction complex composed of E-cadherin, β-catenin, vinculin, and actin cytoskeleton, suggesting that this interaction could sense remote mechanical perturbation across a cell and regulate Piezo1-induced ion gating. Both the aforementioned models cannot be dismissed as it continues to be reported that Piezo1 could be regulated by both mechanisms.

The plasma membrane and the Piezo1 channel itself have been stimulated in various ways, such as poking, stretching, shear stress, and osmotic stress induction, in previous studies (Coste et al., 2010; Ranade et al., 2014; Syeda et al., 2016; Wang et al., 2018). Additionally, Piezo1 was found to be activated by Yoda1 and Jedi1, both of which are Piezo1-specific agonists (Syeda et al., 2015; Wang et al., 2018), although both agonists may not reflect the physiological role of Piezo1 where mechanical force is the native stimuli. Yoda1 can enter the hydrophobic-binding pocket of Piezo1 from the intracellular leaflet of the membrane, which is near the residues 1961–2063 of Piezo1, and effectively lower the mechanical threshold of the channel required for the conformational change of the Piezo1 arm and channel flattening (Botello-Smith et al., 2019). It has been hypothesized that Jedi1 binds to the extracellular side of transmembrane helical units THU1-THU3, and this stimulation passes through two extracellular loops of the blade and beam structure, which opens the Piezo1 channel (Wang et al., 2018). Interestingly, it has been assumed that the Piezo1 activation mechanisms, whether via physical stimulation or chemical treatment, have independent mechanotransduction pathways (Wang et al., 2018; Zhao et al., 2018; Botello-Smith et al., 2019). The Piezo1 R2135A mutant form, which abrogated mechanical opening mediated by membrane stretch, could lead to Ca^2+^ influx in response to Yoda1, and the A2094W mutant form, which severely reduced Yoda1-sensitivity, could trigger inward currents upon pressure (Botello-Smith et al., 2019). Additionally, it was observed in a study that Jedi1 failed to potentiate poking-induced currents in ΔL15-16 or ΔL19-20 Piezo1-expressing cells, whereas Yoda1 successfully induced (Wang et al., 2018; Zhao et al., 2018). Therefore, the Piezo1 channel is opened by a variety of mechanisms, but these mechanisms are not always interrelated.

Various cancer cells change the expression of proteins that are related to calcium ions, such as calcium channels, pumps, exchangers, and binding proteins, to remodel calcium signaling for tumor progression (Stewart et al., 2015; Monteith et al., 2017). Moreover, it has been reported that experimental or pharmacological inhibition of such calcium ion-related proteins influences cancer cell development (Monteith et al., 2017). The elevated levels of Ca^2+^ ions in the cytoplasm can activate cancer cell proliferation *via* cell cycle progression and various cellular signaling pathways. Indeed, calcium plays a significant role in the expression of immediate-early genes, such as *FOS*, *JUN,* and *MYC*, as well as in the phosphorylation of retinoblastoma-associated protein in the G1/S transition, and the activity of various Ca^2+^-dependent transcription factors, including the nuclear factor of activated T-cells, nuclear factor kappa-light-chain-enhancer of activated B cells, and cAMP response element-binding protein (CREB) (Sée et al., 2004; Roderick and Cook, 2008; Prevarskaya et al., 2014). Ca^2+^/calmodulin-dependent protein kinases (CaMKs) and calcineurin are also activated by Ca^2+^, and can facilitate the progression of the cell cycle by regulating cyclin D1 expression and the activity of cyclin-dependent kinases (CDKs) and CREB, a transcription factor that is involved in the G1/S transition, by binding to the cyclin D1 promoter (Tombes et al., 1995; Morris et al., 1998; Khanna and Hosenpud, 1999; Schneider et al., 2002; Kahl and Means, 2004). Calcium and CaMK2 control centrosome duplication and the separation of the duplicated chromosomes to the daughter cells (Roderick and Cook, 2008). There are several cellular pathways that are regulated by calcium which activate cell proliferation; for example, the extracellular signal-regulated kinase 1/2 (ERK 1/2) pathway, which is a downstream effector of Ras, activates activator protein-1 (AP-1) and E26 transformation-specific factor and drives cyclin D1 expression and CDK4 activation (Roderick and Cook, 2008). The Ca^2+^-cAMP-protein kinase A (PKA) pathway also activates CREB (Zhang et al., 2020).

Calcium also promotes cell migration through interactions with various compounds. Calpain, a calcium-dependent protease, cleaves focal adhesion proteins such as integrin, talin, and vinculin, and focal adhesion turnover is upregulated by the phosphorylation of focal adhesion kinase (FAK) *via* CaMK2 and calcineurin (Ridley et al., 2003; Su et al., 2006; Prevarskaya et al., 2011). Myosin light-chain kinase (MLCK) activation by Ca^2+^ phosphorylates myosin light chain (MLC), which results in cell rear-end retraction and allows the cell to migrate toward the front (Yang and Huang, 2005). A conformational change occurs when S100A4, a member of the S100 family of EF-hand calcium-binding proteins, binds to calcium to cause the exposure of its interaction domain, which allows the protein to interact with various cytoskeletal proteins such as actin, non-muscle myosin IIA, non-muscle myosin IIB, myosin heavy chain IIA, and tropomyosin (Kim and Helfman, 2003; Tarabykina et al., 2007). Furthermore, RasGRF1 is activated in a calcium-dependent manner through interaction between its isoleucine-glutamine domain and calmodulin and acts as RacGEF, which activates Rac1 by exchanging Rac1-bound GDP for GTP (Buchsbaum et al., 1996; Zippel et al., 2000; Umeda et al., 2018). The activated Rac1 leads to lamellipodium extension and membrane ruffling by inducing branched actin filament polymerization (Ridley et al., 1992; Connolly et al., 2005). Consequently, calcium and the related signals upregulate cancer cell migration by enhancing focal adhesion turnover, cell rear-end retraction, interaction with cytoskeletal proteins, and branched actin filament polymerization.

**FIGURE 1:**
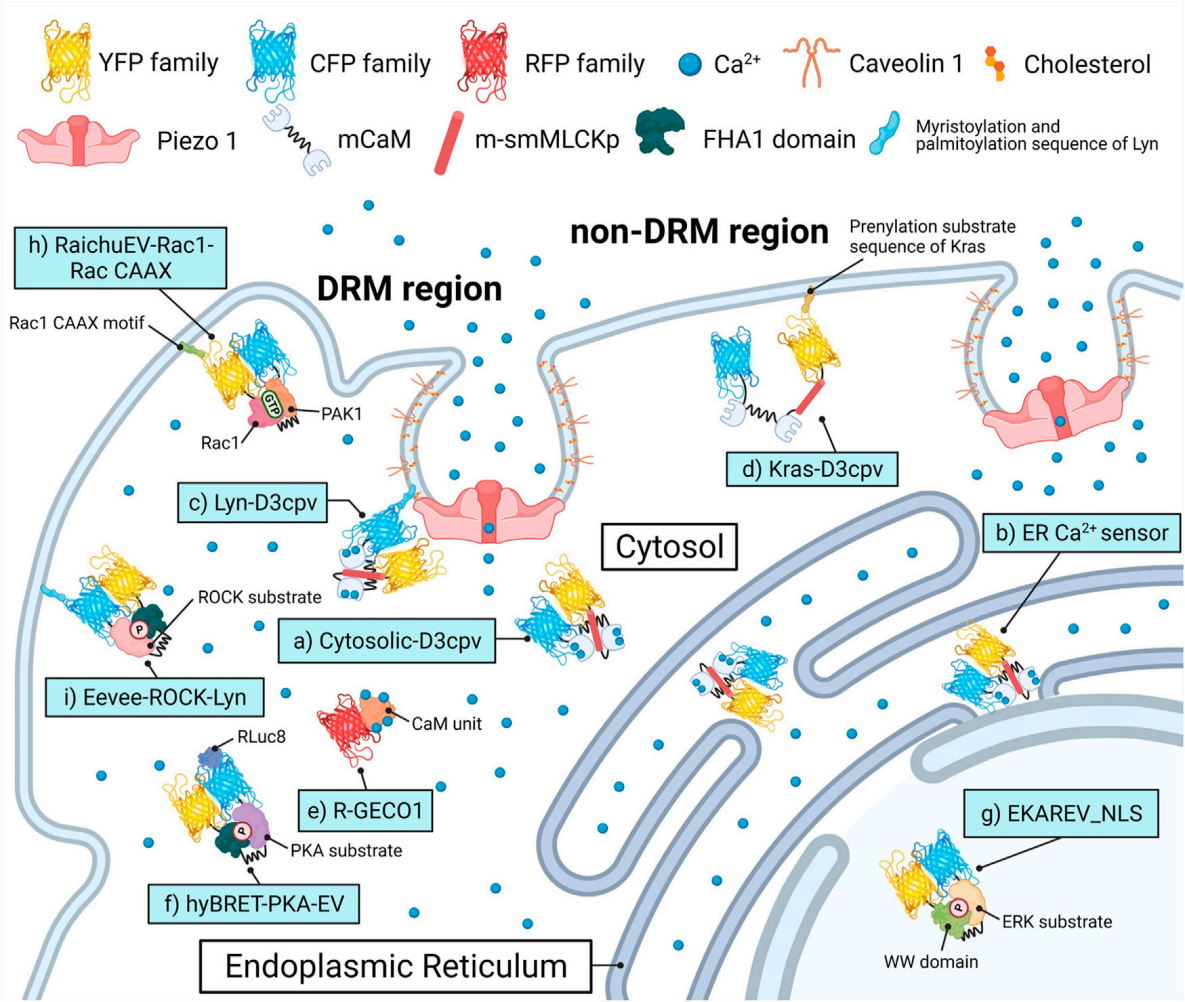
Biosensors and targeting strategies employed in this study. Each of the biosensors visualized the target’s activity in a specific microenvironment and was expressed solely to avoid crosstalk between optical signals of fluorescence proteins. **(A)** Cytosol Ca^2+^ indicator, Cytosolic-D3cpv used in Figures 2, 5. **(B)** Endoplasmic reticulum (ER) Ca^2+^ indicator, ER Ca^2+^ sensor used in Figure 3. **(C)** DRM region Ca^2+^ indicator, Lyn-D3cpv used in Figure 4. **(D)** non-DRM region Ca^2+^ indicator, Kras-D3cpv used in Figure 4. **(E)** Cytosolic Ca^2+^ indicator, R-GECO1 used in Figure 4. **(F)** PKA activity indicator, hyBRET-PKA-EV used in Figure 6. **(G)** ERK activity indicator, EKAREV_NLS used in Figure 6. **(H)** Rac1 activity indicator, RaichuEV-Rac1-Rac CAAX used in Figure 7. **(I)** ROCK activity indicator, Eevee-ROCK-Lyn used in Figure 7. DRM, detergent-resistant membrane. YFP, yellow fluorescent protein. CFP, cyan fluorescent protein. RFP, red fluorescent protein. (m)CaM, (mutated) calmodulin. m-smMLCKp, mutated smooth muscle myosin light chain kinase peptide. FHA1, forkhead-associate 1. PAK1, p21 activated kinase 1. PKA, protein kinase A. ERK, extracellular signal-regulated kinase. Rac1, Rac-GTP complex bound to the P21. ROCK, Rho-associated protein kinase. RLuc8, Renilla luciferase 8.

Although the biological pathways and phenomena that are triggered by Ca^2+^ influx through various channels have been widely studied, the effects of Piezo1 on these phenomena remain unclear. In this study, a variety of biosensors were adopted to visualize various cellular-physiological activities triggered by Piezo1-induced Ca^2+^ flux in the single-cell microenvironment level (Figure 1). We evaluated calcium signal dynamics in the cytoplasm and the endoplasmic reticulum (ER) lumen in response to Yoda1, the plasma membrane microdomain and cytoskeletal structure required for the normal function of Piezo1, and the cellular pathways and membrane ruffling induced by extracellular calcium influx. We found that Piezo1-induced calcium flux upregulated membrane ruffling and cancer cell survival.

## Materials and Methods

### DNA Plasmids and Gene Construction

The detailed information of all plasmids and primer sequences used are listed in Supplementary Tables S1, S2, respectively. Restriction enzyme cloning was performed to construct new plasmids, and the procedure is described in Supplementary Table S3. All PCR products were directly sequenced for genetic confirmation by Macrogen (Republic of Korea).

### Cell Culture and Transfection

The MCF-7, SiHa, and BeWo cell lines were purchased from the Korean Cell Line Bank (KCLB; Seoul, Republic of Korea), and HEK293A and HeLa cell lines were provided by Dr. Jihye Seong (Korea Institute of Science and Technology, Seoul, South Korea). The MCF-7 cells were cultured in RPMI-1640 medium (CM058; GenDEPOT, Barker, TX, United States) supplemented with 10% (v/v) fetal bovine serum (FBS; WB0015; HyClone), 100 U/mL penicillin, 100 μg/ml streptomycin (CA005, GenDEPOT), and 0.01 mg/ml insulin solution from bovine pancreas (I0516, Sigma). The HEK293A, HeLa, and SiHa cells were cultured in Dulbecco’s modified Eagle’s medium (DMEM; CM002, GenDEPOT) containing 10% (v/v) FBS, 100 U/ml penicillin, and 100 μg/ml streptomycin. The BeWo cells were cultured in DMEM/F-12 medium (LM002-08, Welgene) supplemented with 10% (v/v) FBS, 100 U/ml penicillin, and 100 μg/ml streptomycin. The cells were cultured in a humidified incubator with 95% air and 5% CO_2_ at 37°C. The DNA plasmids were transfected into the cells using Lipofectamine 3000 (L3000, Invitrogen) following the manufacturer’s protocol.

### Solutions and Chemicals

To prepare the Ca^2+^-free medium, Hank’s balanced salt solution (HBSS; 14175-095, Gibco) was supplemented with 20 mM hydroxyethylpiperazine ethane sulfonic acid (HEPES; Dojindo), 0.5 mM ethylene glycol-bis(2-aminoethylether)-N,N,N’,N’-tetraacetic acid (EGTA; E3889, Sigma), 1 mM magnesium chloride (MgCl_2_; M8266, Sigma), and 1 mM magnesium sulfate (MgSO_4_; 746452, Sigma) (Kim et al., 2015). To prepare the Ca^2+^ medium, HBSS was supplemented with 20 mM HEPES, 1 mM MgSO_4_, and 1 mM calcium chloride (CaCl_2_; 18230S0301, JUNSEI). The Ca^2+^ medium did not include EGTA, a calcium chelator, or MgCl_2_, a supplemented ion. To represent normal conditions, CO_2_-independent culture medium (18045088, Gibco) containing 0.5% (v/v) FBS, 4 mM l-glutamine (LS002-1, Welgene), 100 U/ml penicillin, and 100 μg/ml streptomycin was used. Yoda1 (SML1558), methyl-β-cyclodextrin (MβCD; 332615), ML-7 (I2764), gadolinium (Ⅲ) chloride hexahydrate (G7535), and thapsigargin (T9033) were purchased from Sigma-Aldrich. Nocodazole (S2775) was purchased from Selleckchem. Cytochalasin D (ab143484) was obtained from Abcam.

### Viability Assay

The WST-8 assay was used to determine the cell viability. The MCF-7 cells were seeded at 8 × 10^3^ cells/well in 96-well plates and incubated for 24 h at 37°C before being treated with an RPMI-containing control (0.5% DMSO) or Yoda1 (0.1–25 μM) for 24 h. After washing, the cells were treated with Cellrix Viability Assay kit (B1007-500, MediFab) in DMEM without phenol red (31053028, Gibco) for 2 h at 37°C. The optical density of the solubilized formazan product was measured using a Glomax Multi + Microplate Multi Reader (9,301–010, Promega, United States) at a wavelength of 450 nm.

### Reverse Transcription-Polymerase Chain Reaction

Total RNA was extracted from MCF-7 cells with TRIsure (BIO-38033, Bioline), and 300 ng of total RNA was used for cDNA synthesis using the Compact cDNA Synthesis kit (SG-CDNAC100, SmartGene) according to the manufacturer’s instructions. To evaluate gene expression levels, RT-PCR was performed with 2× Dye Mixed Hot Start Taq (SG-2XDM-HTaq, SmartGene) following the manufacturer’s protocol. The bands of PCR products were measured densitometrically using ImageJ software version 1.53c (National Institutes of Health; https://imagej.nih.gov/ij). Glyceraldehyde-3- phosphate dehydrogenase (GAPDH) expression was used as a loading control.

### Western Blotting

After washing with cold phosphate-buffered saline (PBS; LB004-02, WELGENE), cells were lysed with the CETi lysis buffer with inhibitors (TLP-121CETi, TransLab) and centrifuged at 13,000 rpm for 15 min at 4°C. Protein concentration was determined using a Pierce BCA Protein Assay Kit (23227, Thermo Scientific) following the manufacturer’s protocol. Each lysate was added to 5× SDS-PAGE Sample Buffer (TLP-102.1, TransLab) and heated at 100°C for 5 min. Proteins (50 μg/lane) were loaded onto 8% SDS-polyacrylamide gel, subjected to electrophoresis, and then transferred to an Immobilon-P PVDF Membrane (IPNH00010, Merck Millipore). The membranes were blocked in 5% (w/v) skim milk in Tris-buffered saline with Tween 20 at pH 7.4 (TBST; TLP-118.1, TransLab) for 1 h 30 min at 20°C. Membranes were incubated with the following primary antibodies in 5% (w/v) skim milk in TBST: anti-PIEZO1 (MBS435119, MyBioSource) diluted 1:500 and anti-GAPDH (HC301, TransGen Biotech) diluted to 1:2000; at 4°C overnight. The membranes were then washed three times with TBST and incubated with horseradish peroxidase-conjugated anti-mouse IgG secondary antibodies (1:2,000; sc-516102, Santa Cruz) in 1% (w/v) skim milk and TBST at 20°C for 60 min. Immunoreactive protein bands were detected using ECL Ottimo (TLP-112.1, TransLab) and measured densitometrically using an iBright FL1500 Imaging System (A44241, Invitrogen) and ImageJ software. GAPDH was used as the loading control.

### Microscopy and Image Acquisition and Analysis

Cells expressing several exogenous proteins were cultured in a confocal dish (100350, SPL Life Sciences) and starved in RPMI-1640 or DMEM containing 0.5% (v/v) FBS for 6 h before imaging. Shortly before the experiment, the cells were washed, and the medium was replaced appropriately according to the experimental conditions. Images were obtained using a Leica DMi8 THUNDER microscope equipped with a scientific complementary metal-oxide-semiconductor (sCMOS) camera (K5, Leica), HC PL APO 40x/1.30 OIL objective lens (11506329, Leica), HC PL APO 100x/1.40–0.70 OIL objective lens (11506220, Leica), CYR71010 filter cube (11525416, Leica), and DFT51010 filter cube (11525418, Leica). Throughout the live imaging, the temperature was maintained at 37°C by an HX Controller (DHC2-0N1C03N, LCI). The detailed filter sets for the fluorescence channels are listed in Supplementary Table S4. The LASX software version 3.6.0. (Leica, https://www.leica-microsystems.com/products/microscope-software/p/leica-las-x-ls/). was used to acquire images and compute the fluorescence emission intensity. A specific region was selected as a region of interest (ROI) to observe the signals and perform quantification. The fluorescence intensity in the background region was selected and quantified to remove the signal from the ROI of the fluorescence channels. The ratio images were displayed in the intensity-modified display mode, where the color of the pixel was determined by the FRET/CFP ratio and red fluorescence protein (RFP) intensity. Quantified values were analyzed using the GraphPad Prism (version 7.0.0) software for Windows (GraphPad Software; https://www.graphpad.com/). The control groups showed that the imaging environments, including temperature, pH, and drug treatment process, did not disturb the cell physiology.

### Spatiotemporal Image Correlation Spectroscopy Analysis

Spatiotemporal Image Correlation Spectroscopy (STICS) analysis was used to determine the direction and velocity of the GFP-cortactin localized in the lamellipodia of cells using the ImageJ plugin STICS map jru v2 (Stowers Institute for Medical Research in Kansas City; https://research.stowers.org/imagejplugins/), which actualizes a method developed by Hebert et al. After visualizing the membrane ruffling by live-cell imaging, the LASX software corrected the background of the fluorescence images and exported successive images in the AVI format. The ImageJ software converted the video files to grayscale and 32-bit images and executed the STICS map jru v2. For one experimental group, we analyzed 12 independent cells and selected one ROI per cell. The ROI consisted of 9 (3 × 3) particles. The obtained flow mapping images were overlaid with gray-scaled GFP-cortactin images, and the velocity values were analyzed statistically.

### Statistical Analysis

All results are expressed as mean ± standard error of the mean (SEM) with GraphPad Prism 7.0.0. Statistical analyses were performed using unpaired two-tailed *t*-test to determine the statistical significance of the differences between two groups. We considered the *p*-values at *<0.05, **<0.01, ***<0.001, and ****<0.0001 to be statistically significant.

## Results

### Piezo1-Mediated Extracellular Ca^2+^ Influx and Endoplasmic Reticulum-Stored Ca^2+^ Release by Yoda1

**FIGURE 2:**
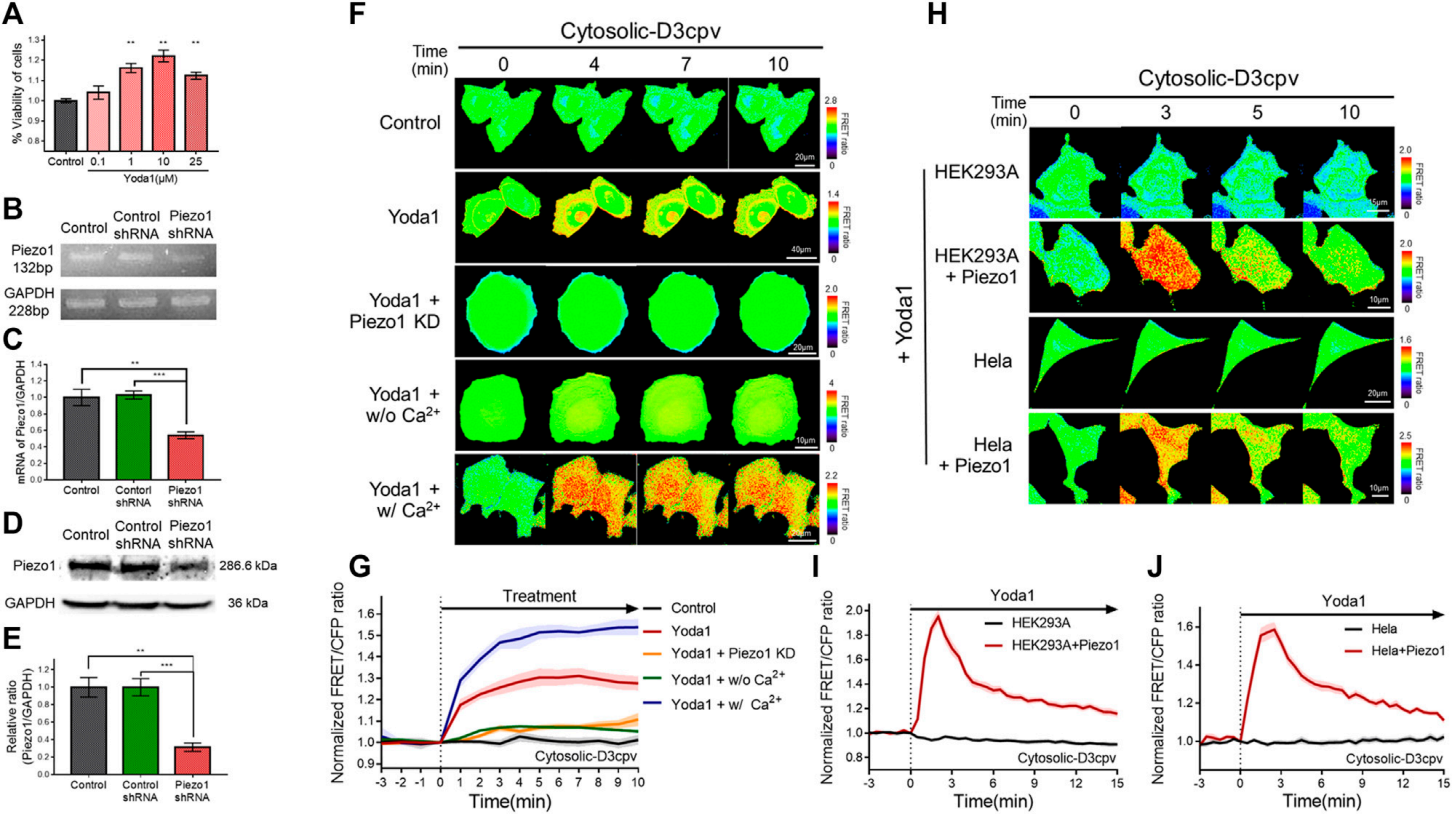
Piezo1-mediated extracellular Ca^2+^ influx induced by Yoda1 in various cell lines. **(A)** Viability of MCF-7 cells exposed to control [0.2% (v/v) DMSO] and Yoda1 (0.1–25 μM) for 24 h, measured using the WST-8 assay. The bar graphs describe mean values of cell viability with error bars indicating the standard error of the mean (S.E.M) (*n* = 4, **p < 0.01, student *t*-test). **(B,D)** Piezo1 expression in MCF-7 cells with or without control/Piezo1 shRNA as analysed by **(B)** RT-PCR and **(D)** western blotting. GAPDH was used as an internal control. **(C,E)** The bar graphs describe the cumulative densitometric analysis of **(C)** RT-PCR and **(E)** western blot bands normalized to the GAPDH. Data are presented as the means ± S.E.M (*n* = 4, **p < 0.01 and ***p < 0.001, student *t*-test). **(F,H)** Time-lapse FRET images of the Cytosolic-D3cpv in **(F)** MCF-7 cells, **(H)** HEK293A and HeLa cells introduced with or without exogenous Piezo1. MCF-7 cells were exposed to control [0.2% (v/v) DMSO] and Yoda1 (1 μM) dissolved in CO_2_-independent culture medium (Yoda1 group), Ca^2+^-free medium (Yoda1 + w/o Ca^2+^ group), and Ca^2+^ medium (Yoda1 + w/Ca^2+^ group), respectively. **(F)** Yoda1 + Piezo1 KD group and **(H)** HEK293A and HeLa cells were incubated in CO_2_-independent culture medium. The color scale bars represent the range of the FRET/CFP emission ratio determined using the biosensor. Hot and cold colors indicate high and low Ca^2+^ concentration, respectively. **(G,I,J)** The time courses represent the average of the normalized FRET/CFP emission ratio changes of cytosolic-D3cpv in **(G)** MCF-7, **(I)** HEK293A, and **(J)** HeLa cells. The lines are mean values of normalized emission ratios, with diluted colors indicating the S.E.M (*n* = 7).

Yoda1 was used to activate the Piezo1 channel in order to visualize Ca^2+^ influx through the channel and the associated cell biological activity. We performed a viability assay before the live-cell imaging to determine the appropriate treatment concentration and the cytotoxicity of Yoda1 (Figure 2A). All experimental groups that were administered Yoda1 did not show cell death and survived to a higher extent compared to the control group; 0.1–10 μM Yoda1 increased cell viability in a dose-dependent manner, and the increase in viability was relatively low but still higher than that of the control at 25 μM (Figure 2A). Thus, treatment with Yoda1 enhanced cell viability, and a concentration range of 1–5 μM Yoda1 was applied to the cells in this study. Next, reverse transcription-polymerase chain reaction (RT-PCR) and Western blotting were performed to confirm the function of Piezo1 shRNA used in this study (Figures 2B–E). Piezo1 shRNA lowered the mRNA level of Piezo1 to 54% and the protein level to 31%, whereas the cells transfected with the control shRNA did not show a decrease in the mRNA and protein levels, revealing that the introduction of Piezo1 shRNA silenced the channel specifically (Figures 2B–E).

We transfected cytosolic-D3cpv, a FRET-based cytosolic Ca^2+^ indicator, into MCF-7 cells which express Piezo1 endogenously (Figures 2F,G) to detect Yoda1-induced extracellular Ca^2+^ influx. The biosensor had been designed to report an increased FRET/CFP ratio when the calcium level in the cytoplasm increases. We observed an increase in cytosolic Ca^2+^ levels when Yoda1 was added to the cells expressing cytosolic-D3cpv that were incubated in a CO_2_-independent culture medium (Yoda1 group). Next, Yoda1 was administered to cells co-transfected with cytosolic D3cpv and Piezo1 shRNA to verify that calcium influx was dependent on Piezo1, but not other calcium-permeable channels, evidenced by a significant decrease in the FRET/CFP ratio (Yoda1 + Piezo1 KD group). To confirm that the increase in the FRET/CFP ratio was specifically caused by extracellular Ca^2+^ influx, we utilized a Ca^2+^-free medium (Yoda1 + w/o Ca^2+^ group) to create a Ca^2+^-free environment outside the cell. A significant reduction in the ratio was also observed. We reaffirmed that the decrease in the FRET/CFP ratio was specifically due to calcium, and not due to any other ions unintentionally excluded, using cells incubated in the Ca^2+^ medium (Yoda1 + w/Ca^2+^ group), and observed a higher FRET/CFP ratio in these cells compared to that the Yoda1 group. Collectively, our results showed that Yoda1 induced extracellular Ca^2+^ influx by specifically stimulating Piezo1, and the FRET/CFP ratio obtained by cytosolic-D3cpv was influenced specifically by calcium and represented the change in Ca^2+^ levels in the cytoplasm (Figures 2F,G).

We next visualized the cytoplasmic calcium levels of various cell lines treated with Yoda1 to study whether the Yoda1-induced dynamics of extracellular Ca^2+^ influx are different in different cell lines. We co-transfected the biosensor and Piezo1-tdTomato into these cell lines and observed FRET/CFP ratio change after Yoda1 treatment (Figures 2H–J), while both the native HEK293A and HeLa cell lines transfected with cytosolic D3cpv did not show FRET/CFP ratio change. Although HEK293 and Hela cells endogenously express functional Piezo1 (McHugh et al., 2010; Dubin et al., 2017), the expression levels may not be sufficient to respond to 1 μM Yoda1. The results obtained confirmed that Yoda1 specifically activated Piezo1. In contrast to the MCF-7 cells, both the HEK293A and HeLa cell lines transfected with Piezo1 revealed a relatively short duration of FRET/CFP ratio peak and faster recovery of the ratio to the baseline (Figures 2I,J). We administrated Yoda1 to SiHa and BeWo cell lines, which express Piezo1 endogenously, and observed that the SiHa cells showed relatively slow recovery of the calcium level to the baseline, similar to the observation with the MCF-7 cells. However, the calcium signal dynamics in the BeWo cells were similar to those of the HEK293A and HeLa cells transfected with exogenous Piezo1 (Supplementary Figure S2). Thus, these results suggested that the calcium influx caused by Yoda1-treated Piezo1 and its dynamics differed depending on the cell line.

**FIGURE 3:**
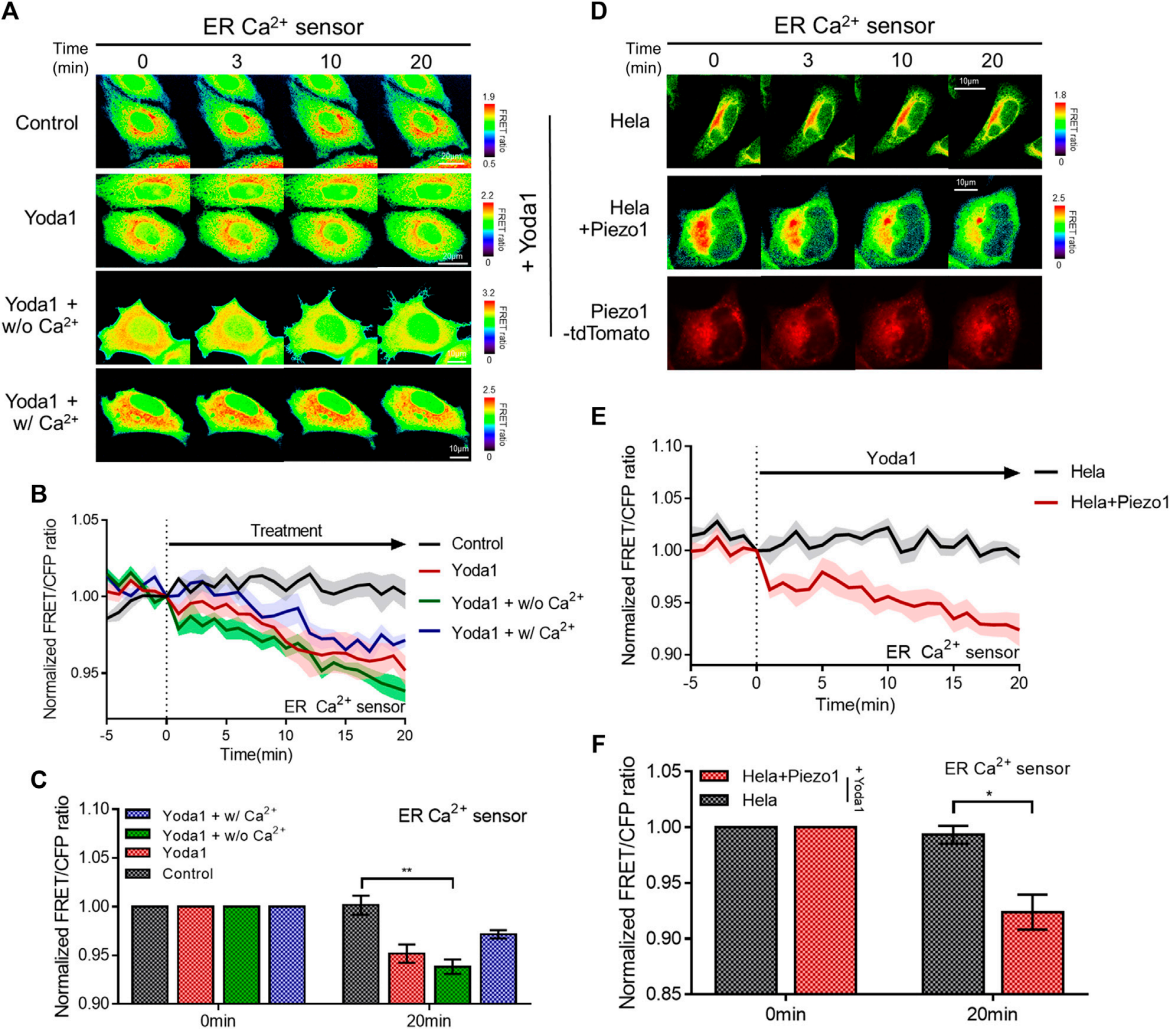
Piezo1-dependent ER-stored Ca^2+^ release in response to Yoda1. **(A,D)** Time-lapse FRET images of the ER Ca^2+^ sensor in **(A)** MCF-7 and **(D)** HeLa cells. The cells were exposed to control [0.2% (v/v) DMSO] and Yoda1 (1 μM) dissolved in CO_2_-independent culture medium (Yoda1 group), Ca^2+^-free medium (Yoda1 + w/o Ca^2+^ group), and Ca^2+^ medium (Yoda1 + w/Ca^2+^ group), respectively. The color scale bars represent the range of the FRET/CFP emission ratio determined using the biosensor. Hot and cold colors indicate high and low Ca^2+^ concentration, respectively. **(D)** The red fluorescence protein images confirmed the expression of Piezo1-tdTomato. **(B,E)** The time courses represent the average of the normalized FRET/CFP emission ratio changes of the ER Ca^2+^ sensor. The lines are mean values of the normalized emission ratios, with diluted colors indicating the S.E.M (*n* = 7). **(C,F)** The bar graph describes the mean values of the normalized FRET/CFP emission ratios of the ER Ca^2+^ sensor at the described time with error bars indicating the S.E.M (*n* = 7, *p < 0.05 and **p < 0.01, Student’s *t*-test).

We determined whether the calcium concentration in the ER changed when cells were treated with Yoda1 since a slight increase in cytosolic calcium levels was observed when the cells were exposed to Yoda1 under Ca^2+^-free conditions (Figure 2G). The ER Ca^2+^ sensor, which has a calreticulin signal sequence (CRsig) and ER retention sequence (KDEL) at the N terminus and C terminus of cytosolic-D4cpv, respectively (Kim et al., 2017), was expressed in the MCF-7 cells, and live imaging was conducted, which revealed that ER calcium levels decreased when the cells were exposed to Yoda1 (Figures 3A–C). Interestingly, the Yoda1 + w/o Ca^2+^ group, which was in an extracellular calcium-free environment, released more calcium than the Yoda1 and Yoda1 + w/Ca2+ groups in extracellular calcium. Therefore, we assumed that calcium restoration in the ER does not occur properly if cells were not supplied with calcium from the extracellular region. Additionally, the results suggest that Yoda1-induced ER-stored calcium release did not require extracellular calcium. In addition, we administrated Yoda1 after gadolinium (Gd^3+^) pre-treatment, which reduces the extracellular calcium influx mediated by Piezo1 (Deivasikamani et al., 2019). We hypothesized that cells exposed to Gd^3+^ release more ER stored Ca^2+^, which is mediated by ER membrane-located Piezo1, in response to Yoda1 than “Yoda1” experimental group, although there is calcium in the extracellular region. In groups administrated with Gd^3+^, 1 μM Yoda1 led to a higher FRET ratio decrease than the Yoda1 group (Supplementary Figure S3). To confirm the Piezo1-indeced ER-stored Ca^2+^ dynamics, we treated thapsigargin (TG), which depletes Ca^2+^ in ER, before Yoda1 treatment. Interestingly, Yoda1 resulted in immediate calcium influx into ER in the presence of extracellular calcium (Supplementary Figure S4). We assume that Piezo1-mediated extracellular Ca^2+^ influx increases cytosolic calcium concentration and the ion flow into ER via routes except for Piezo1. However, Piezo1-mediated ER calcium influx occurred slowly in the absence of extracellular calcium (Supplementary Figure S4). Recently, it was reported that Piezo1 is at various subcellular organelles, in particular mitochondria, and the channel activation increases cytosolic Ca^2+^ concentration (Liao et al., 2021). Mitochondria have a relatively higher Ca^2+^ concentration than the cytoplasm (Arnaudeau et al., 2001). Therefore, these results could suggest that Yoda1 activates Piezo1 located at mitochondria and leads to mitochondria-stored Ca^2+^ efflux, and the ion was slowly introduced into ER deficient in calcium through other pathways except Piezo1. Next, we monitored the ER-stored Ca^2+^ level of the HeLa cell line to examine whether ER-stored calcium release depended on Piezo1 expression and found that extracellular Ca^2+^ influx was not observed in response to Yoda1 (Figures 3D–F). HeLa cells expressing the ER Ca^2+^ sensor alone did not trigger a decrease in the FRET/CFP ratio. However, the cells co-transfected with the biosensor and exogenous Piezo1 showed ER-stored calcium release (Figures 3D–F). Therefore, Yoda1 induced ER calcium release by activating Piezo1, and the degree of ER calcium release was affected by extracellular Ca^2+^ levels.

### Intact Caveolin, Cholesterol, and Various Cytoskeletal Structures Are Required for the Normal Function of Piezo1

The detergent-resistant membrane (DRM), known as a lipid raft, is a membrane microdomain that contains an amount of sphingolipid and cholesterol and acts as a scaffold for many different signaling pathways because a variety of integral membrane proteins are embedded in it, such as insulin-like growth factor-I receptor and phosphoinositide 3-kinases (Huo et al., 2003; Mollinedo and Gajate, 2015). Caveolae, a subset of DRM characterized by omega-or cup-shaped membrane microdomains, contains caveolins that line the membrane and stabilize the caveolae structure (Pelkmans et al., 2004; Schlörmann et al., 2010). Lipid rafts and caveolar microdomains play essential roles in modulating calcium signals because these structures contain various key proteins, which are associated with Ca^2+^, such as STIM1, CaMK2, PKC, and PLC (Pani and Singh, 2009). In particular, cholesterol and multiple signaling molecules in the microdomain interact with diverse ion channels and regulate the function and membrane localization of channels (Levitan et al., 2010). It has been reported that methyl-β-cyclodextrin (MβCD), a cholesterol-depletion agent, completely reduced the β2-adrenergic stimulation of Cav1.2 channels (Balijepalli et al., 2006; Tsujikawa et al., 2008) and suppressed the activity of mechanosensitive channels in human leukemia K562 cells (Morachevskaya et al., 2007). TRPM7 was reported to localize in fractions associated with caveolae in bradykinin-stimulated cells, suggesting that TRPM7-caveolae-lipid raft association may facilitate the localization of TRPM7 to cell membrane receptors (Yogi et al., 2009). Components of membrane microdomain also play a role in Piezo1 activation and inactivation; margaric acid inhibits Piezo1 activation, and polyunsaturated fatty acids modulate channel inactivation (Romero et al., 2019). In addition, cholesterol depletion with MβCD caused a shift in the midpoint activation pressure of Piezo1, increased channel latency, and slowed channel inactivation (Ridone et al., 2020).

**FIGURE 4:**
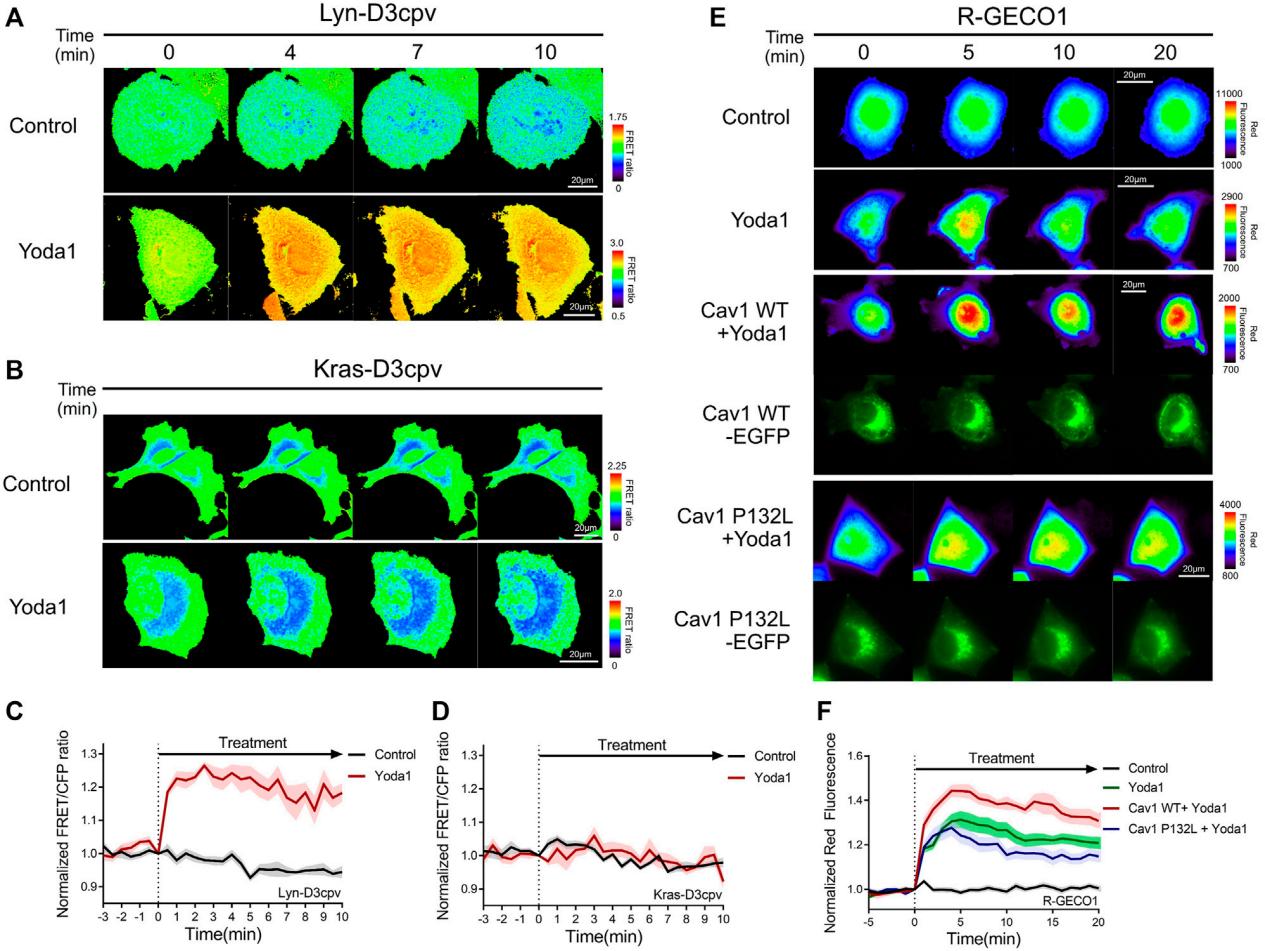
Detergent-resistant membrane (DRM) and caveolin were required for normal Piezo1 function. **(A,B)** Time-lapse FRET images of the **(A)** Lyn-D3cpv and **(B)** Kras-D3cpv in MCF-7 cells. **(E)** Time-lapse red fluorescence protein (RFP) emission ratio images of the R-GECO1 in MCF-7 cells transfected with Cav1 WT and Cav1 P132L. The cells were exposed to the control [0.2% (v/v) DMSO] and Yoda1 (1 μM) dissolved in CO_2_-independent culture medium. The color scale bars represent the range of the **(A,B)** FRET/CFP or **(E)** RFP emission ratios determined using the biosensor. Hot and cold colors indicate high and low Ca^2+^ concentrations, respectively. The images of green fluorescence channel confirmed the expression of the intended caveolin-1 constructs **(C,D,F)** The time courses represent the average of the normalized **(C,D)** FRET/CFP emission ratio changes of **(C)** Lyn-D3cpv and **(D)** Kras-D3cpv, and **(F)** RFP emission ratio changes of R-GECO1. The lines are mean values of the normalized emission ratios, with diluted colors indicating the S.E.M [**(C,D)** *n* = 7, **(F)** *n* = 20 for control and Cav1 P132L + Yoda 1, and *n* = 14 for Yoda1 and Cav1 WT + Yoda1].

Live-cell imaging was performed to study the location and functions of Piezo1, and whether this channel requires lipids and membrane microdomain structures/for its normal function. We employed two distinct types of FRET-based Ca^2+^ biosensors tethered at DRM and non-DRM microdomains (Kim et al., 2018) (Figures 4A–D) to assess the variation of Piezo1-induced calcium influx with the type of membrane microdomains. Lyn-D3cpv is used to visualize the calcium signal of the DRM region by using the myristoylation and palmitoylation sequence of Lyn kinase at the N-terminal sequence of cytosolic D3cpv. Kras-D3cpv, which has a prenylation substrate sequence of Kras at the C-terminus of cytosolic D3cpv, was utilized to observe the change in calcium concentration in the non-DRM region. We observed a change in the FRET/CFP ratio when the cells transfected with Lyn-D3cpv were exposed to Yoda1, indicating the occurrence of Ca^2+^ influx at the DRM region. However, the FRET/CFP ratio did not change in the group transfected with Kras-D3cpv (Figures 4A–D). Next, we investigated whether mutated caveolin-1 influences Yoda1-induced Piezo1 Ca^2+^ gating function. MCF-7 cells were co-transfected with R-GECO1, a red fluorescent genetically encoded Ca indicator (Zhao et al., 2011), and caveolin-1 wild-type (Cav1 WT) or caveolin 1 P132L (Cav1 P132L), a mutant that has oligomerization defects and accumulates in the ER or Golgi apparatus and does not support caveolae formation (Hayer et al., 2010) (Figures 4E,F). In response to Yoda1, the expression of wild-type caveolin 1 in the plasma membrane (Cav1 WT + Yoda1 group) induced a relatively higher Piezo1-mediated Ca^2+^ influx than the Yoda1 group, which only expresses R-GECO1 (Figures 4E,F). The cells expressing Cav1 P132L showed a relatively lower calcium influx than that in the Cav1 WT group post-treatment with Yoda1 (Figures 4E,F).

**FIGURE 5:**
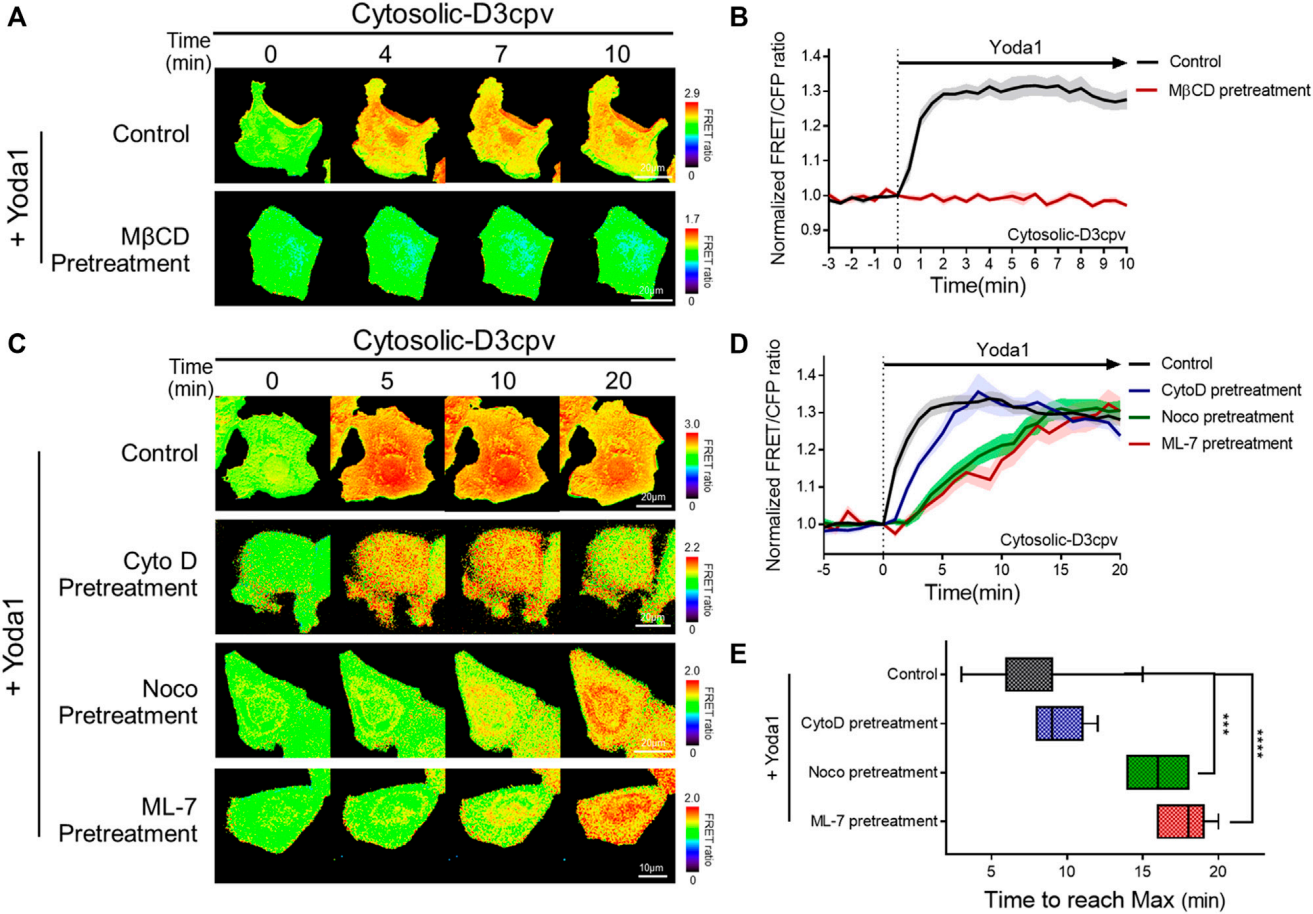
Intactness of cholesterol, microtubules, and actomyosin structures plays an important role in the normal function of Piezo1. **(A,C)** Time-lapse FRET images of the cytosolic-D3cpv in untreated MCF-7 cells and cells pretreated with **(A)** MβCD, **(C)** cytochalasin D (CytoD), nocodazole (Noco), and ML-7. All cells were exposed to Yoda1 (1 μM) dissolved in CO_2_-independent culture medium. The color scale bars represent the range of the FRET/CFP emission ratio detected using the biosensor. Hot and cold colors indicate high and low Ca^2+^ concentrations, respectively. **(B,D)** The time courses represent the average of the normalized FRET/CFP emission ratio changes of the cytosolic-D3cpv in **(B)** MβCD, **(D)** CytoD, Noco, and ML-7 pretreatment groups. The lines are mean values of normalized emission ratios, with diluted colors indicating the S.E.M (*n* = 7). **(E)** The box plots describe the time taken to reach the maximum value of the FRET/CFP ratio in the control, CytoD, Noco, and ML-7 pretreatment groups with whiskers representing minimum and maximum values, respectively. (*n* = 7, ***p < 0.001 and ****p < 0.0001, Student *t*-test).

Live cell imaging was then performed to verify whether the integrity of cholesterol in the lipid raft is required for the normal function of Piezo1. We administered Yoda1 after the cholesterol of cells expressing cytosolic D3cpv was depleted by using 5 mM MβCD for 1 h. No cytosolic calcium influx (Figures 5A,B) was observed. In previous work, which used relatively low concentrations of MβCD, the cholesterol depletion interfered with the dynamics of 5 μM Yoda1-induced calcium signaling (Chong et al., 2021). Our results showed that Piezo1-mediated Ca^2+^ influx dynamics by 1 μM Yoda1 could absolutely be destroyed by 5 mM MβCD pretreatment. These results are consistent with earlier reports that the compressive stress-induced calcium influx via Piezo1 is reduced following the reduction of Cav1 expression and MβCD treatment (Mingzhi et al., 2019). Collectively, our results show that Piezo1 mainly induced calcium influx in the DRM region, and the integrity of the caveolin and the cholesterol constituting the lipid raft is required for the normal function of Piezo1.

There are three major cytoskeleton structures connected to the cell plasma membrane: Actin filaments, microtubules, and actomyosin (Sitarska and Diz-Muñoz, 2020). These structures support the membrane and play an essential role in mechanotransduction (Gittes et al., 1993; Ananthakrishnan and Ehrlicher, 2007; Clark et al., 2007; Salbreux et al., 2012; Murrell et al., 2015; Morley et al., 2016; Pandya et al., 2017; Yan et al., 2018). We hypothesized that changes in the cytoskeletal support affect the function of Piezo1, which detects mechanical stress from the membrane and extracellular microenvironments. Cells expressing cytosolic D3cpv were pretreated with cytochalasin D (CytoD), which disrupts actin microfilaments (Brenner and Korn, 1980); nocodazole (Noco), which binds to β-tubulin and interferes with the polymerization of microtubules (Mejillano et al., 1996); and ML-7, an MLCK inhibitor (Saitoh et al., 1987) for 30 min, 1 h, and 1 h respectively, and exposed to Yoda1 (Figures 5C–E) to examine Piezo1-mediated calcium signals in cells where specific single cytoskeleton structure was compromised. We observed that CytoD pretreatment had a negligible effect on calcium influx dynamics. However, cells with microtubule disruption induced by Noco (Noco group) and ML-7 group having abnormal actomyosin activity took a longer time to reach the maximum FRET/CFP ratio value compared to that in the control and CytoD groups (Figures 5C–E). Collectively, our results suggest that the integrity of the microtubules and actomyosin is required for the calcium signal dynamics caused by Yoda1-induced Piezo1 activation to function properly. Cells implicates the cytoskeleton in the dynamics of the Ca^2+^ signaling not the magnitude, thus they are for all intents and purposes dispensable for the Yoda-1 mediated activation of Piezo1.

### Yoda1 Promoted PKA, ERK, Rac1, and ROCK Activity in an Extracellular Ca^2+^-And Piezo1-Dependent Manner

**FIGURE 6:**
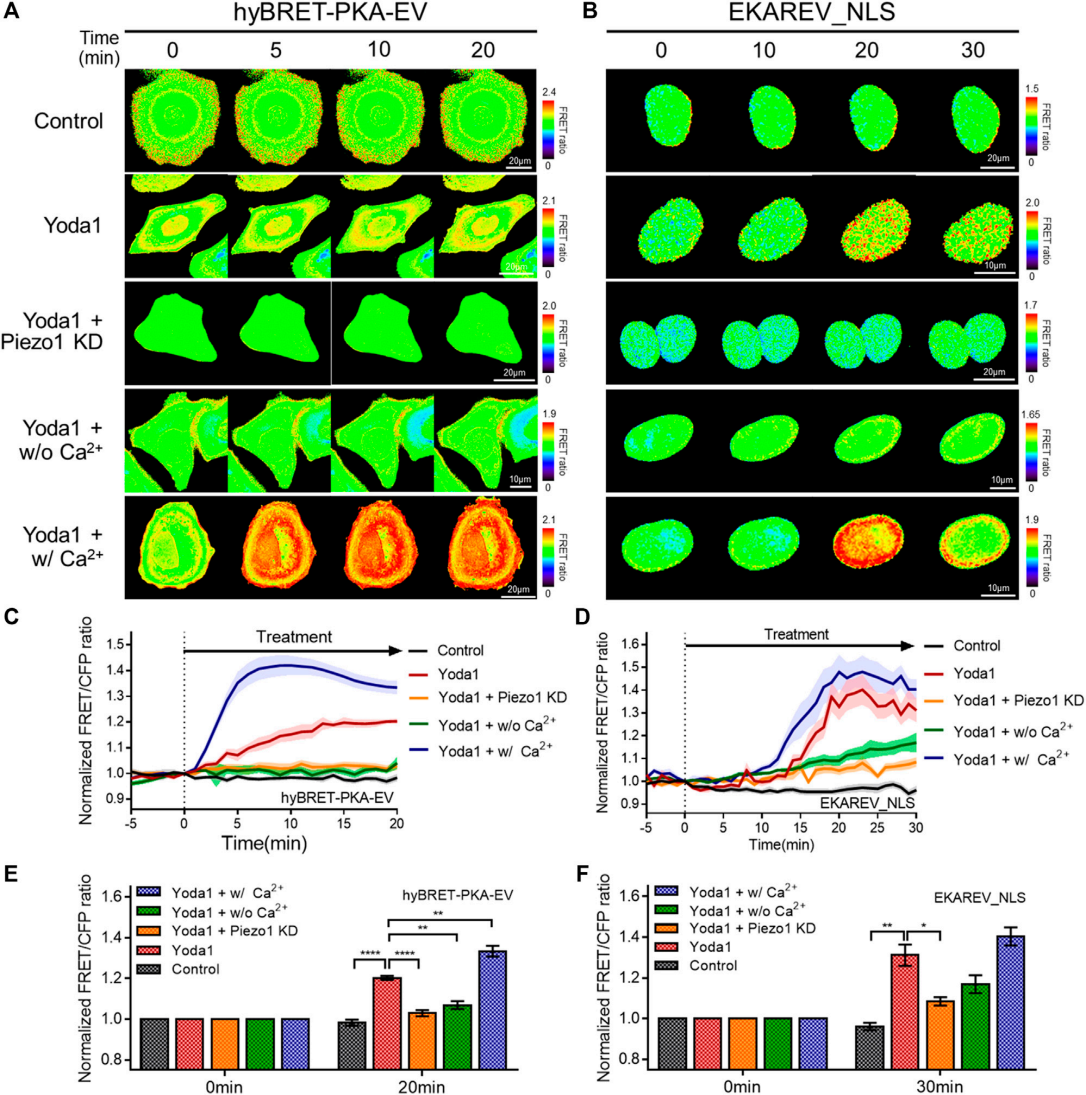
Effects of Piezo1-induced cytosolic calcium increase on PKA and ERK activity. **(A,B)** Time-lapse FRET images of the **(A)** hyBRET-PKA-EV and **(B)** EKAREV_NLS in MCF-7 cells. The cells were exposed to the control [0.2% (v/v) DMSO] and Yoda1 (5 μM for hyBRET-PKA-EV and 1 μM for EKAREV_NLS) dissolved in CO_2_-independent culture medium (Yoda1 group), Ca^2+^-free medium (Yoda1 + w/o Ca^2+^ group) and Ca^2+^ medium (Yoda1 + w/Ca^2+^ group), respectively. The Yoda1 + Piezo1 KD group was incubated in CO_2_-independent culture medium. The color scale bars represent the range of the FRET/CFP emission ratio determined using the biosensor. Hot and cold colors indicate high and low **(A)** PKA and **(B)** ERK activity, respectively. **(C,D)** The time courses represent the average of the normalized FRET/CFP emission ratio changes of **(C)** hyBRET-PKA-EV and **(D)** EKAREV_NLS. The lines are mean values of normalized emission ratios, with diluted colors indicating the S.E.M (*n* = 7). **(E,F)** The bar graph describes the mean values of the normalized FRET/CFP emission ratios of **(E)** hyBRET-PKA-EV and **(F)** EKAREV_NLS at the indicated times with error bars indicating the S.E.M (*n* = 7, *p < 0.05, **p < 0.01 and ****p < 0.0001, Student’s *t*-test).

We investigated the biological pathways that are activated by Piezo1-mediated extracellular calcium influx, since increased cytosolic calcium has an effect on various cell signaling pathways. We used the hyBRET-PKA-EV and EKAREV_NLS biosensors, which are designed to show increased FRET/CFP ratios by the activation of PKA and ERK, respectively, to examine whether increased cytosolic calcium promotes the PKA and ERK signaling pathways that enhance cell survival and proliferation (Figures 6A–F) (Komatsu et al., 2011; Komatsu et al., 2018). Yoda1-induced activation of PKA and ERK was observed in the cells of the Yoda1 group incubated in a CO_2_-independent culture medium, but this increase was significantly reduced in the Piezo1 KD group. We observed a decrease in the FRET/CFP ratio in the absence of extracellular calcium. We then reaffirmed whether the decrease in the FRET/CFP ratio resulted only from calcium by using a medium containing Ca^2+^ (Yoda1 + w/Ca^2+^ group), and observed a higher FRET/CFP ratio compared to that observed in the Yoda1 group (Figures 6A–F).

**FIGURE 7:**
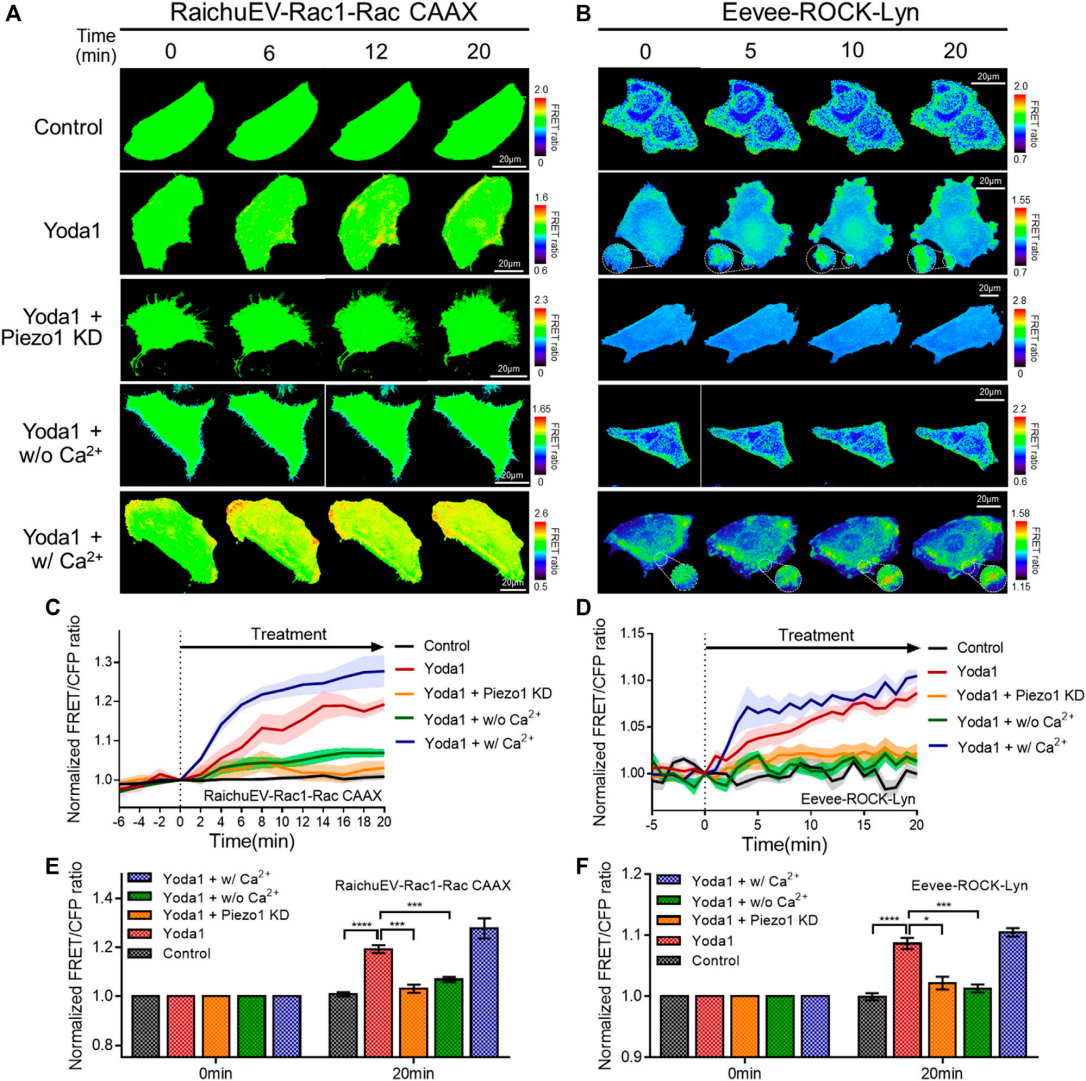
Extracellular calcium influx through Piezo1 increases Rac1 and ROCK activity. **(A,B)** Time-lapse FRET images of the **(A)** RaichuEV-Rac1-Rac CAAX and **(B)** Eevee-ROCK-Lyn in MCF-7 cells. The cells were exposed to control [0.2% (v/v) DMSO] and Yoda1 (1 μM for RaichuEV-Rac1-Rac CAAX and 5 μM for Eevee-ROCK-Lyn) dissolved in CO_2_-independent culture medium (Yoda1 group), Ca^2+^-free medium (Yoda1 + w/o Ca^2+^ group), and Ca^2+^ medium (Yoda1 + w/Ca^2+^ group), respectively. The Yoda1 + Piezo1 KD group was incubated in a CO_2_-independent culture medium. The color scale bars represent the range of the FRET/CFP emission ratio determined using the biosensor. Hot and cold colors indicate high and low **(A)** Rac1 and **(B)** ROCK activity, respectively. **(C,D)** The time courses represent the average of the normalized FRET/CFP emission ratio changes of **(C)** RaichuEV-Rac1-Rac CAAX and **(D)** Eevee-ROCK-Lyn. The lines are mean values of normalized emission ratios, with diluted colors indicating the S.E.M (*n* = 7). **(E,F)** The bar graph describes the mean values of the normalized FRET/CFP emission ratios of **(E)** RaichuEV-Rac1-Rac CAAX and **(F)** Eevee-ROCK-Lyn at the indicated times with the error bars indicating the S.E.M (*n* = 7, *p < 0.05, ***p < 0.001 and ****p < 0.0001, Student’s *t*-test).

Subsequently, the RaichuEV-Rac1-Rac CAAX and Eevee-ROCK-Lyn biosensors were employed to visualize the activity of Rac1 and ROCK, which is a downstream effector of RhoA. The forementioned biosensors detect an increased FRET/CFP ratio in response to Rac1 and ROCK activation, respectively (Komatsu et al., 2011; Li et al., 2017) (Figures 7A–F). We observed that Yoda1 promoted the activity of Rac1 and ROCK, and decreased the expression of Piezo1 by shRNA reduced the response of these two effectors to Yoda1. The FRET/CFP ratio reduced in the Yoda1 + w/o Ca^2+^ group when extracellular calcium was absent; however, the cells incubated in the Ca^2+^ medium showed the highest Rac1 and ROCK activation (Figures 7A–F). Intriguingly, we observed that Yoda1 slightly activated the ERK, Rac1, and ROCK in Yoda1 + Piezo1 KD group (Figures 6D,F and Figures 7C–F). It has been previously reported that Yoda1 slightly induced the activation of ERK and Akt independent of Piezo1 (Dela Paz and Frangos, 2018), which is consistent with our results. Nevertheless, Piezo1 and calcium play a significant role in the activation of ERK, Rac1 and ROCK because the presence of extracellular calcium and the integrity of Piezo1 expression caused greater activation of cellular signals in all the Yoda1 treatments. Therefore, extracellular calcium influx through Piezo1 upregulates PKA, ERK, Rac1, and ROCK activities which have the potential to promote cancer cell survival, proliferation, and migration.

### Extracellular Ca^2+^ Influx *via* Piezo1 and Downstream Effectors Regulates Membrane Ruffling

**FIGURE 8:**
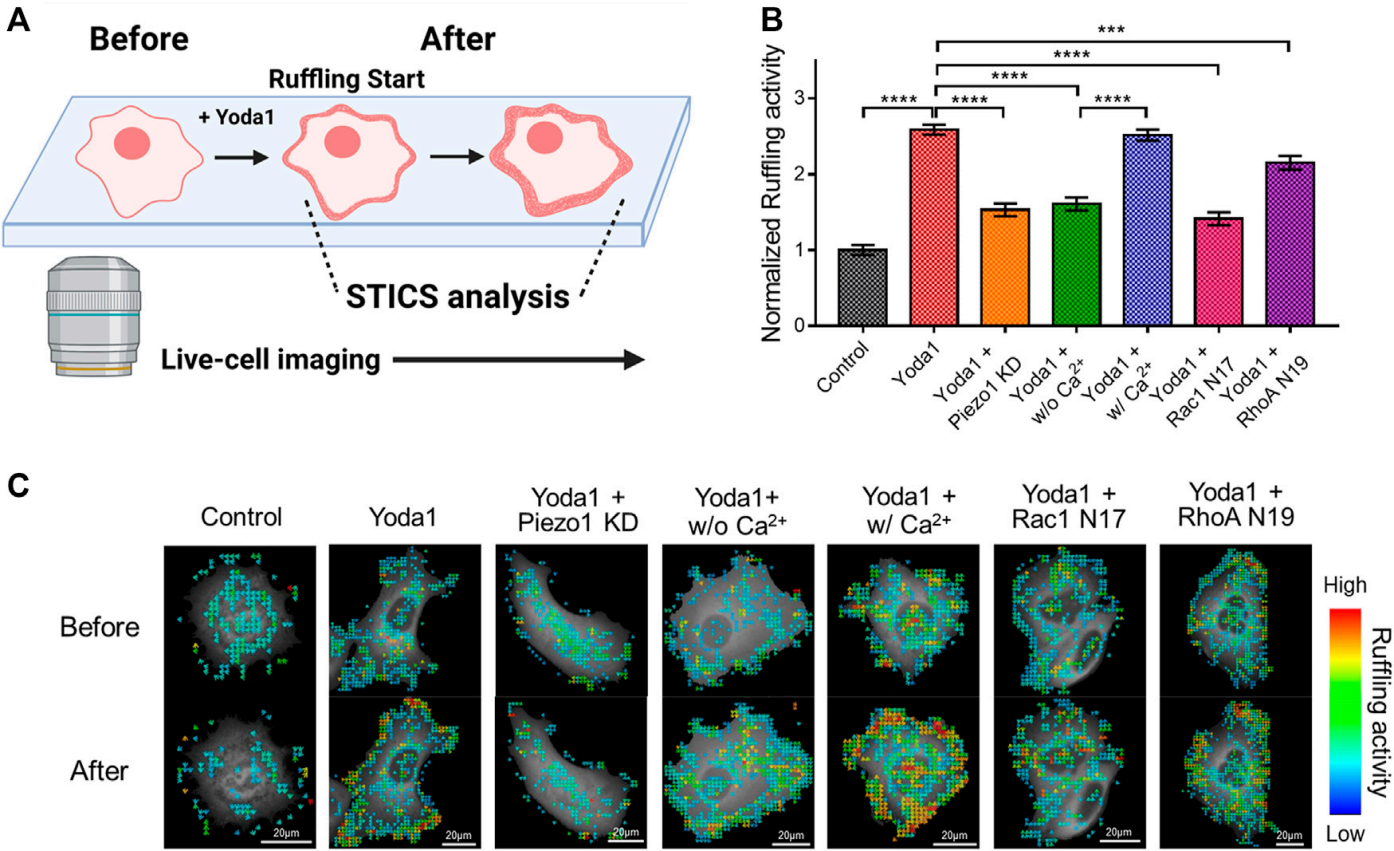
Effects of Piezo1-induced extracellular calcium influx on membrane ruffling. **(A)** A schematic illustration explaining the procedure for analyzing the membrane ruffling with STICS. Live-cell imaging was used to visualize membrane ruffling, which occurs briefly after Yoda1 treatment, and STICS analyzed the videos, or a series of images, showing the movement. The illustration was created using BioRender (https://biorenders.com/) **(B)** The bar graph describes the mean values of the normalized ruffling activity with error bars indicating the S.E.M (the number of analyzed particles = 108, ***p < 0.001 and ****p < 0.0001, Student’s *t*-test). The cells were treated with the control [0.2% (v/v) DMSO] and Yoda1 (1 μM) dissolved in CO_2_-independent culture medium (Yoda1 group), Ca^2+^-free medium (Yoda1 + w/o Ca^2+^ group), and Ca^2+^ medium (Yoda1 + w/Ca^2+^ group), respectively. The Yoda1 + Piezo1 KD, Rac1 N17, and RhoA N19 groups were incubated in a CO_2_-independent culture medium. **(C)** The flow mapping images represent the velocity of the GFP-cortactin particles expressed in MCF-7 cells. The color scale bars represent the range of the ruffling activity calculated by STICS. Hot and cold colors indicate high and low ruffling activity, respectively.

Cells extend protrusions toward desired spaces and form new focal adhesions for migration (Mayor and Etienne-Manneville, 2016). Specifically, lamellipodia, a sheet-like membrane protrusion, is mainly found at the leading edge and results from branched actin filament polymerization (Krause and Gautreau, 2014; Mayor and Etienne-Manneville, 2016). Migrating cells generate lamellipodia by activating the Rac-WAVE/SCAR-Arp2/3 signaling axis. The actin-rich dendritic network appears to look like the cell membrane is forming hair-like ruffles when viewed from the extracellular regions, which is called membrane ruffling (Krause and Gautreau, 2014; Isogai et al., 2015). It has been reported that the store-operated Ca^2+^ entry (SOCE) complex composed of STIM1 and ORAI1 plays an important role in membrane ruffling (Lopez-Guerrero et al., 2017). As shown in Figure 7, Piezo1-induced extracellular calcium influx upregulated the activity of Rac1, which promotes branched actin filament polymerization, and ROCK, which plays a role in ROCK-mediated actin cytoskeleton rearrangement, and rear-end retraction. Since Piezo1 activation-induced membrane ruffling remains poorly understood, we explored this phenomenon using spatiotemporal image correlation spectroscopy (STICS), which analyses the velocity of particles in live imaging data (Hebert et al., 2005; Yi et al., 2011; Hartzell et al., 2016) (Figure 8). We transfected MCF-7 cells with GFP-tagged cortactin, a membrane ruffling marker, to observe membrane ruffling activity. Cortactin, a substrate of Src, mediates cell shaping, membrane protrusion, membrane ruffling, and lamellipodia by contributing to actin cytoskeleton rearrangement (Wu and Parsons, 1993; Weed and Parsons, 2001; Ammer and Weed, 2008). We visualized real-time cell membrane ruffling after Yoda1 treatment, and the velocity values of the fluorescence particles in the ruffling region were derived with STICS; these values were named “ruffling activity” (Figure 8A). We observed active membrane ruffling in the Yoda1 group (Supplementary Video S2) following Yoda1 administration to the cells incubated in the CO_2_-independent culture medium, but the ruffling was significantly reduced in the Piezo1 KD group (Figures 8B,C) (Supplementary Video S3). The cells incubated in calcium-free conditions showed decreased cell membrane ruffling; however, ruffling activity in the Yoda1 + w/Ca^2+^ group was the same as that in the Yoda1 group (Supplementary Videos S4, S5). We then introduced Rac1 N17 and RhoA N19, a dominant-negative construct of Rac1 and RhoA, respectively, into each group to identify the downstream effectors that regulate the membrane ruffling induced by Piezo1-mediated extracellular calcium influx. As expected, the cells introduced with dominant-negative RhoA exhibited more active membrane ruffling than the Rac1 N17-introduced cells (Figures 8B,C) (Supplementary Videos S6, S7) post-Yoda1 treatment. We conjectured that continuous and active membrane ruffling in the Yoda1 and Yoda1 + w/Ca^2+^ groups required the presence of extracellular calcium and the intactness of Piezo1 and the downstream effectors (Supplementary Videos S1–7). Collectively, these results show that Piezo1-induced extracellular calcium influx results in membrane ruffling, a precursor of cell migration, and this ruffling is regulated to a greater extent by Rac1 compared to that by RhoA.

## 4 Discussion

Various aspects of Piezo1 have been studied over the years, including the structure of the channel, mechanogating mechanism, activation dynamics, its interaction with surrounding proteins and agonists, physiological functions, and associated diseases (Coste et al., 2010; Coste et al., 2012; Lukacs et al., 2015; Gudipaty et al., 2017; Jin et al., 2017; Wang et al., 2018; Botello-Smith et al., 2019; Wang et al., 2020). In this study, we investigated Yoda1-treated MCF-7 cells, which express Piezo1 endogenously, and explored Piezo1-induced Ca^2+^ flux, cellular signaling, and cellular requirements for the normal function of the channel. Additionally, membrane ruffling mediated by Ca^2+^ influx and Piezo1 was also examined.

Yoda1 administration triggered the decrease in the ER calcium sensor FRET/CFP ratio, indicating the release of ER-stored calcium (Figure 3). Since Yoda1-induced ER-stored calcium release remains poorly understood, we suggest two potential theories to explain this phenomenon. First, Piezo1 located in the ER is activated by Yoda1, resulting in calcium efflux. During the synthesis of most membrane proteins in the ER that are located in the plasma membrane, some parts of the proteins are inside the ER or vesicles and then the proteins turn towards the extracellular region in the plasma membrane. In other word, the parts always face the exoplasmic space (i.e., the lumen of the ER, vesicles and cell exterior) (Harvey et al., 2008). It has been demonstrated that a variety of calcium-permeable ion channels, which are mainly in the plasma membrane and induce extracellular calcium influx, are also located in the ER membrane and mediate ER-stored calcium release (Takeshima et al., 2015). Previous studies showed that Piezo1 is also present in the ER (Satoh et al., 2006; McHugh et al., 2010). Recently, it has been reported that Piezo1 located in ER membrane triggers ER-stored Ca^2+^ release (Liao et al., 2021). In addition, the patch of negatively charged residues 2393-DEEED-2397 of Piezo1, placed above the extracellular fenestration sites, has an important role in the selection of cations over anions, suggesting that cations might enter the ion-conducting pathway through the “extracellular” fenestration sites (Zhao et al., 2018). Therefore, we could also conclude that cations go into the central pore through the “exoplasmic space,” the lumen of the ER. Accordingly, we suggest a model that Piezo1 is located in the ER-lipid bilayer, and the top part of the channel which usually faces the extracellular region in the plasma membrane might face the inside of ER, and Piezo1 could trigger ER-stored Ca^2+^ release. Since Yoda1 binds to the Piezo1-hydrophobic binding pocket near the membrane’s intracellular leaflet (Botello-Smith et al., 2019), the agonist can effectively approach the binding site, which is exposed to the cytosol and is found in both the plasma membrane and ER. Therefore, we hypothesized that Yoda1 treatment activated Piezo1 and induced calcium release in the ER. In this work, we mainly examined and validated this hypothesis. Second, the Ca^2+^ influx mediated by Piezo1 activates ER-located calcium efflux channels and receptors. As illustrated in Figure 6, PKA was upregulated in a Piezo1-induced calcium-dependent manner. Ca^2+^-calmodulin signaling promotes cAMP levels by upregulating adenylyl cyclase, and PKA, its downstream effector, activates ryanodine receptors (RYRs) and inositol 1,4,5-trisphosphate receptors (IP_3_Rs), which mediate ER-stored calcium release (Reiken et al., 2003; Taylor, 2017). Hence, Piezo1-induced calcium influx in itself creates an environment that can cause ER-stored calcium release.

As shown in Figures 5C–E, the cells pretreated with Noco and ML-7 showed different dynamics of Yoda1-induced calcium signaling than those observed in the control group. Intriguingly, the maximum values of the FRET/CFP ratio were similar among all groups; Noco and ML-7 delayed the time to reach the maximum ratio value (Figures 5D,E). The plasma membrane, where various transmembrane and membrane-bound proteins are embedded in the lipid bilayer, is supported by the cytoskeleton complex (Sitarska and Diz-Muñoz, 2020). Components of the cytoskeleton, including microtubules and actomyosin, are key intracellular structures that support membrane mechanics and play an essential role in mechanotransduction (Gittes et al., 1993; Ananthakrishnan and Ehrlicher, 2007; Murrell et al., 2015; Morley et al., 2016; Yan et al., 2018). The microtubules, whose polymerization was interrupted by nocodazole, are rigid structures in cells and play a significant role in maintaining cell shape and polarized pseudopodial activity (Rodionov et al., 1993). Upon the acetylation of α-tubulin, which is a component of the microtubule, the stiffness of the plasma membrane is increased, and cells require more force to trigger mechanosensitive channels (Morley et al., 2016; Yan et al., 2018). In addition, TRPV1, a non-selective cation channel, binds to a network of subcortical microtubules *via* cytoplasmic tubulin-binding motifs in mammalian osmo-sensory neurons (Prager-Khoutorsky et al., 2014). Therefore, microtubules play an important role in maintaining cellular elasticity and mechanotransduction. It was reported that the microtubule stabilizer paclitaxel increased Piezo2-mediated mechanically activated currents, but these currents were reduced by vincristine, a microtubule destabilizer, suggesting that microtubules are involved in Piezo2 mechanotransduction (Chang and Gu, 2020). Thus, the results of the previous study showed similar trends to our results, showing that microtubule disruption by nocodazole results in altered Piezo1 function. The cell cortex contains membrane-bound actomyosin, which is composed of myosin II and its substrate, F-actin (Sitarska and Diz-Muñoz, 2020). The interaction between myosin II and actin filaments generates a contractile force that controls the cell shape and plays a role in mechanotransduction (Clark et al., 2007; Murrell et al., 2015; Pandya et al., 2017). ML-7 is a selective inhibitor of MLCK, which phosphorylates MLC and potentiates contractility in actomyosin (Saitoh et al., 1987). Therefore, we assume that pre-treatment with ML-7 reduces the contractile force of actomyosin, alters intracellular tension and plasma membrane elasticity, and changes the activity of Piezo1, which senses mechanical stimulus from the local membrane or ambient cellular microenvironment.

## 5 Conclusion

**FIGURE 9:**
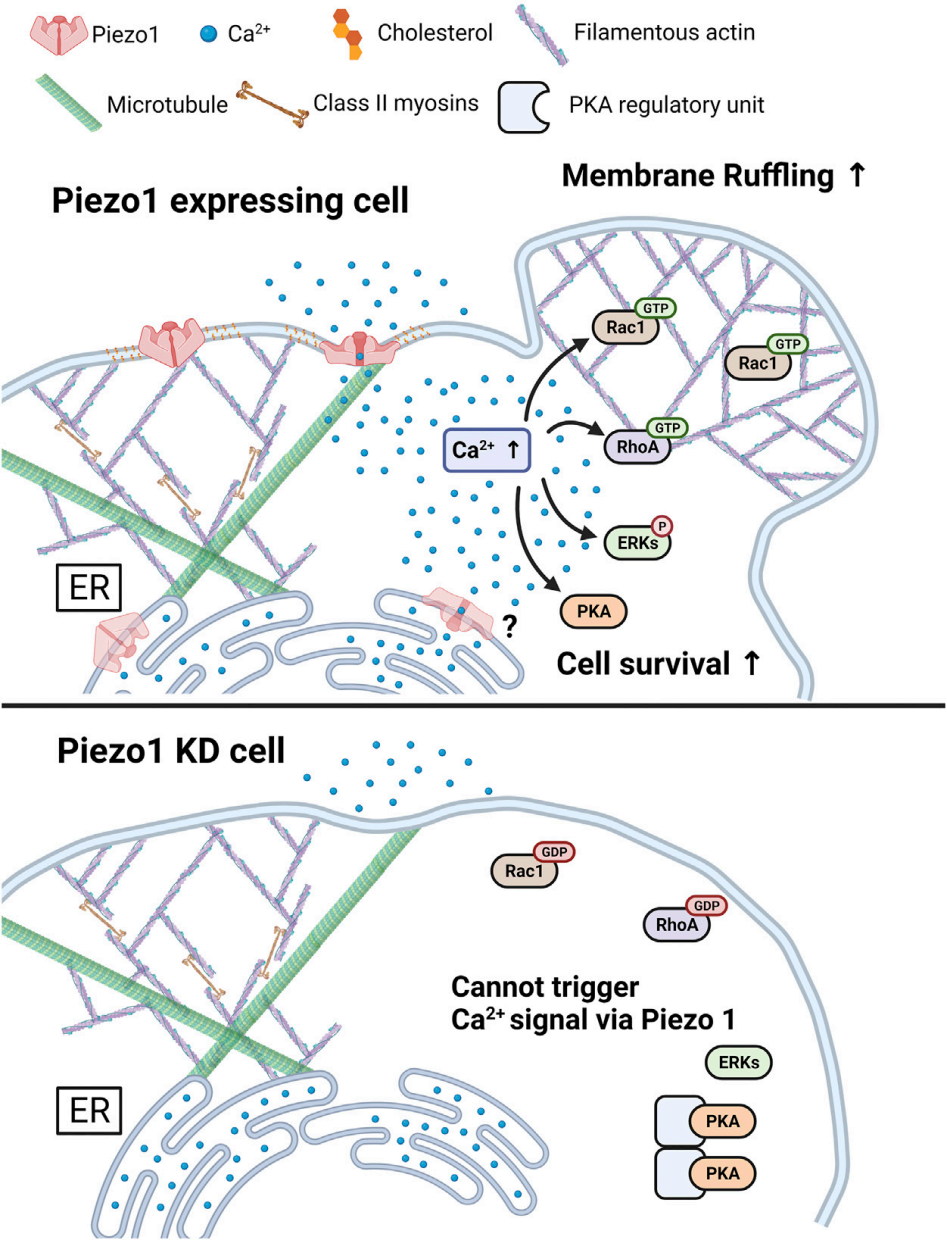
A graphical summary describes the effects of Piezo1-mediated Ca^2+^ flux. The increase in cytosolic Ca^2+^ levels induced by Piezo1 activates PKA, ERK, RhoA, and Rac1, and promotes cell survival and membrane ruffling. For the normal functioning of Piezo1, the integrity of caveolin, cholesterol, and cytoskeletal structure such as microtubules and actomyosin structures was required. The Piezo1 KD cell inhibits the triggering of activation of Ca^2+^ signal, following cell survival and membrane ruffling induced by Piezo1. The figure was created using BioRender.

In conclusion, we found that Yoda1-induced Piezo1 activation increased cytosolic calcium levels *via* extracellular calcium influx and release of ER-stored calcium. The intactness of the caveolin, cholesterol and cytoskeletal structures are required for Piezo1 to function normally. Furthermore, Yoda1 treatment induced the activation of PKA, ERK, Rac1, and ROCK, and membrane ruffling in a Ca^2+^-and Piezo1-dependent manner. Taken together, our results indicate that Piezo1 induces calcium flux-upregulated cell survival and membrane ruffling (Figure 9). These data provide a novel insight that cancer cells expressing Piezo1 might use the channel to promote cell survival and migration; therefore, Piezo1 could be an important target for treating such cancer cells.

## Data Availability

The raw data supporting the conclusion of this article will be made available by the authors, without undue reservation.
