# Supplementary figures and images for: Förster Resonance Energy Transfer-Based Single-Cell Imaging Reveals Piezo1-Induced Ca2+ Flux Mediates Membrane Ruffling and Cell Survival
Source: Front Cell Dev Biol. 2022 May 13;10:865056. doi: 10.3389/fcell.2022.865056 (PMC9136143; doi:10.3389/fcell.2022.865056)

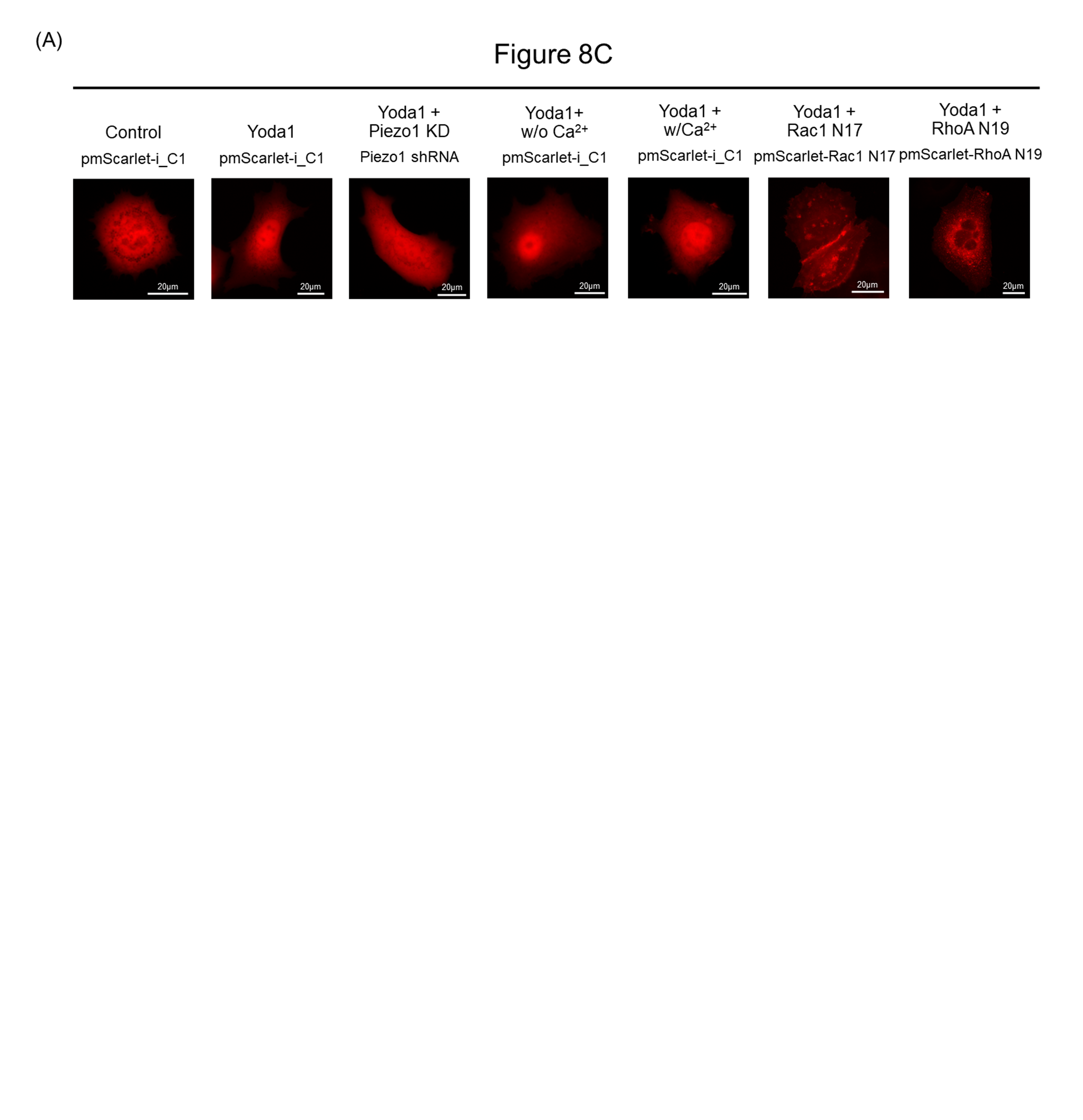

Supplement: Supplementary file 1 [file Image6.TIF]

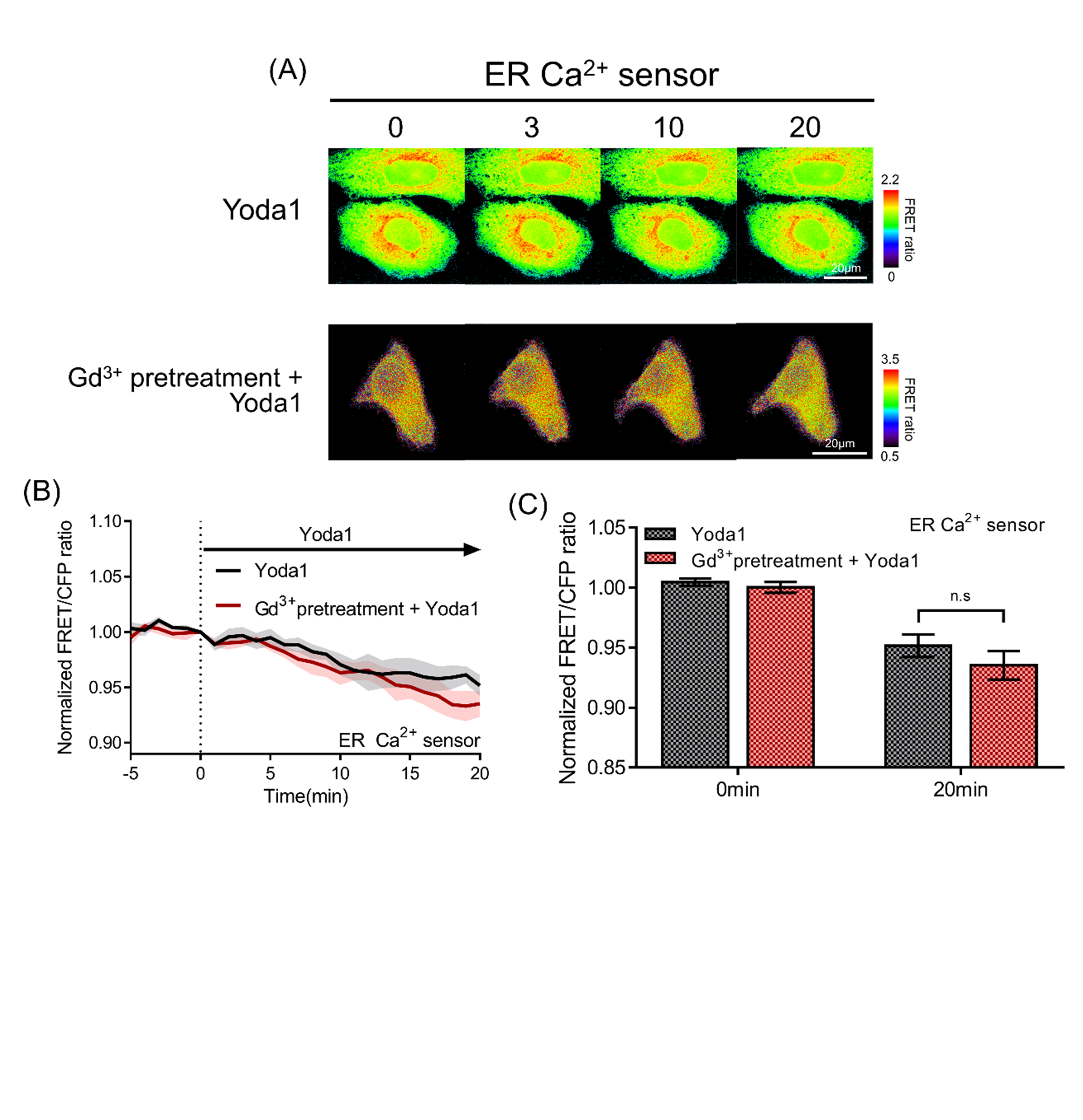

Supplement: Supplementary file 3 [file Image3.TIF]

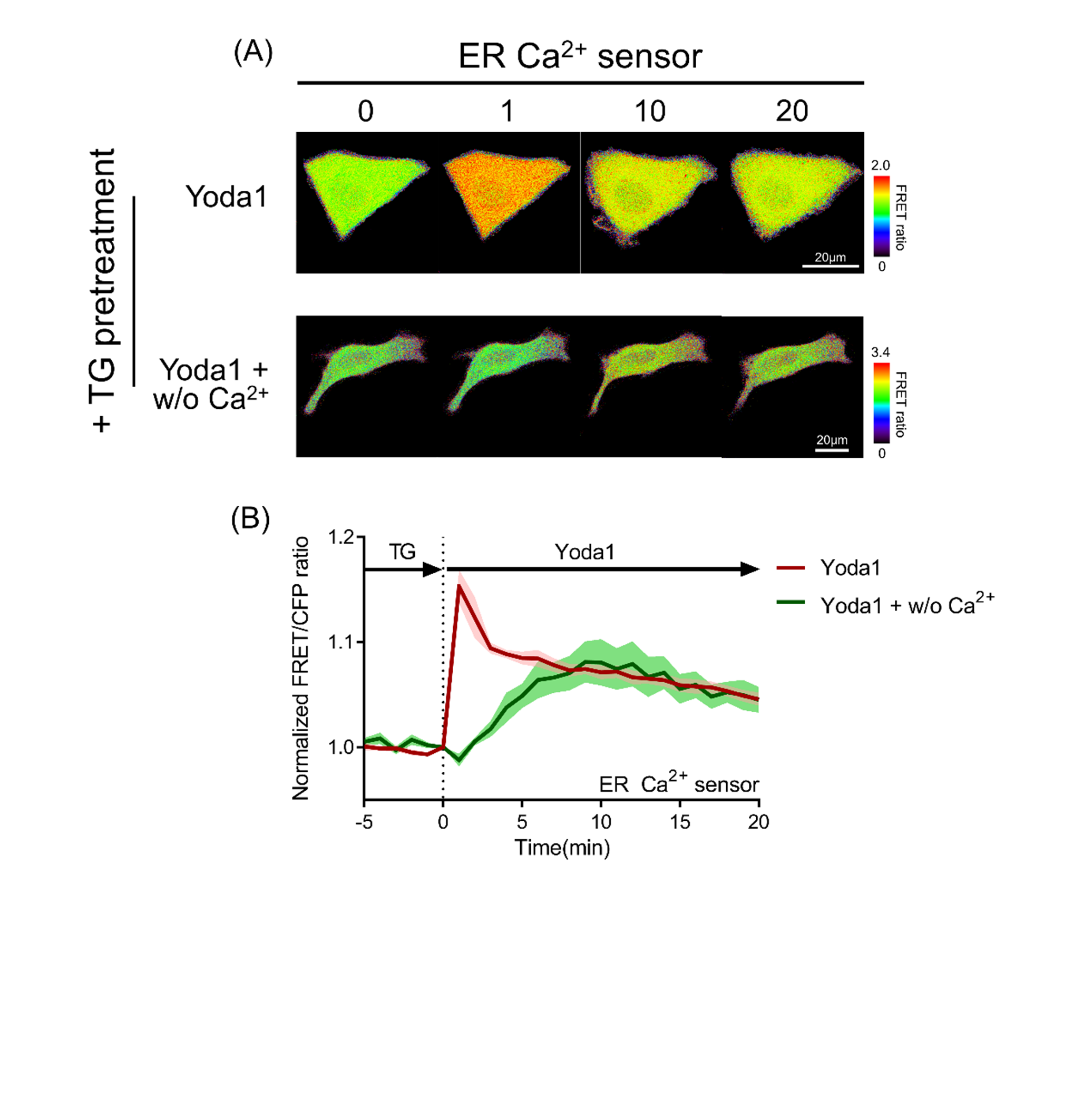

Supplement: Supplementary file 4 [file Image4.TIF]

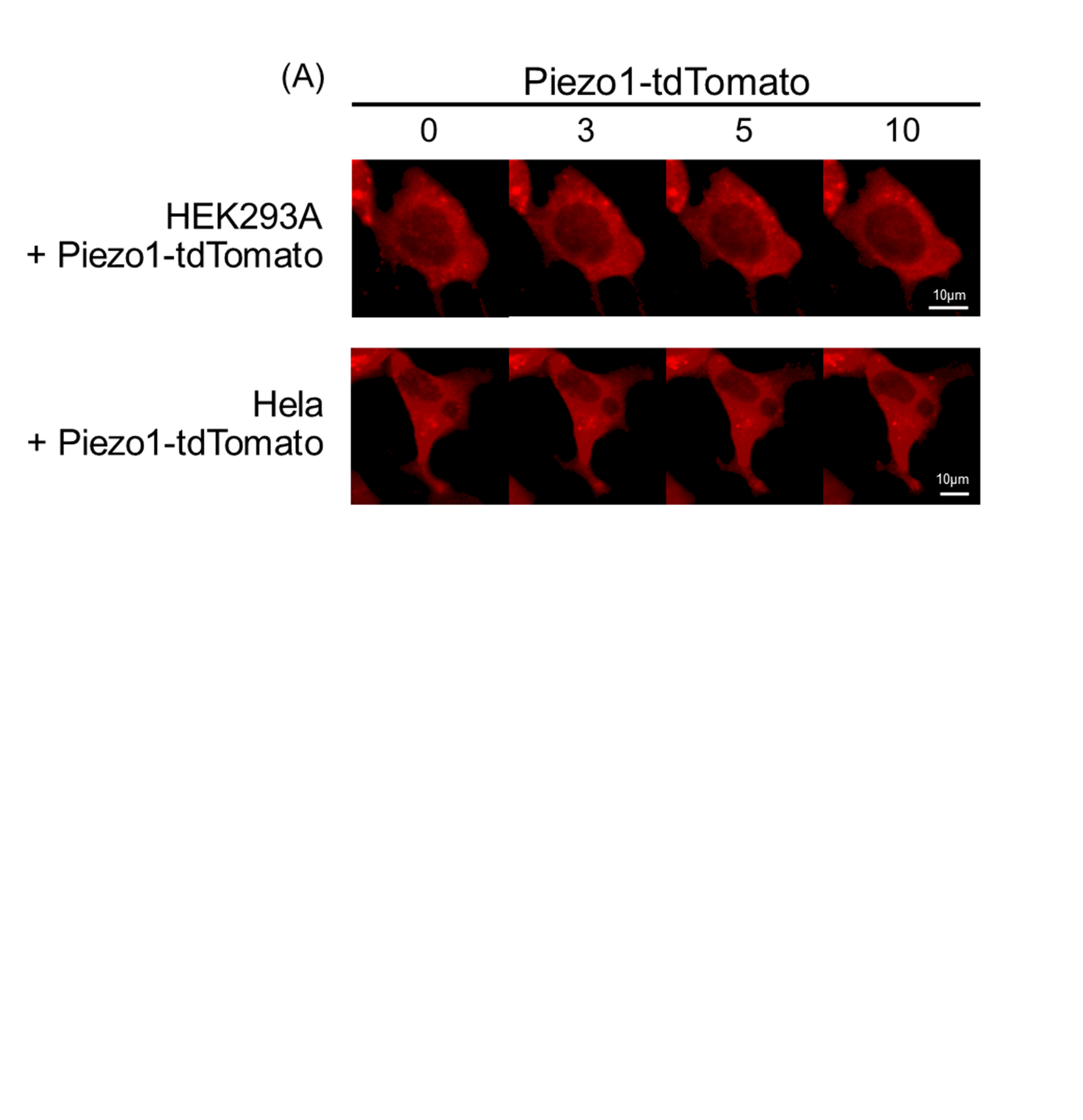

Supplement: Supplementary file 5 [file Image2.TIF]

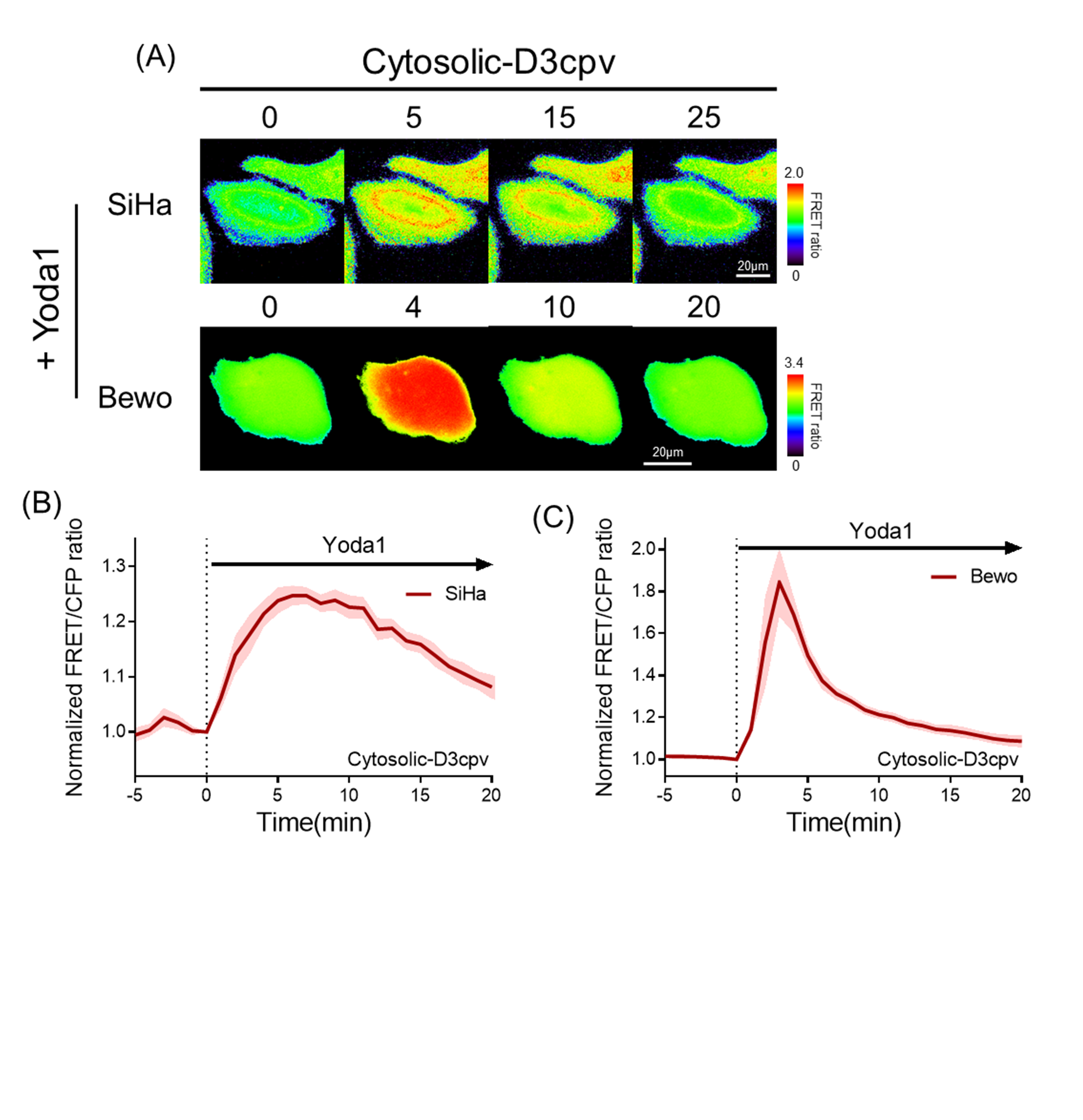

Supplement: Supplementary file 6 [file Image1.TIF]

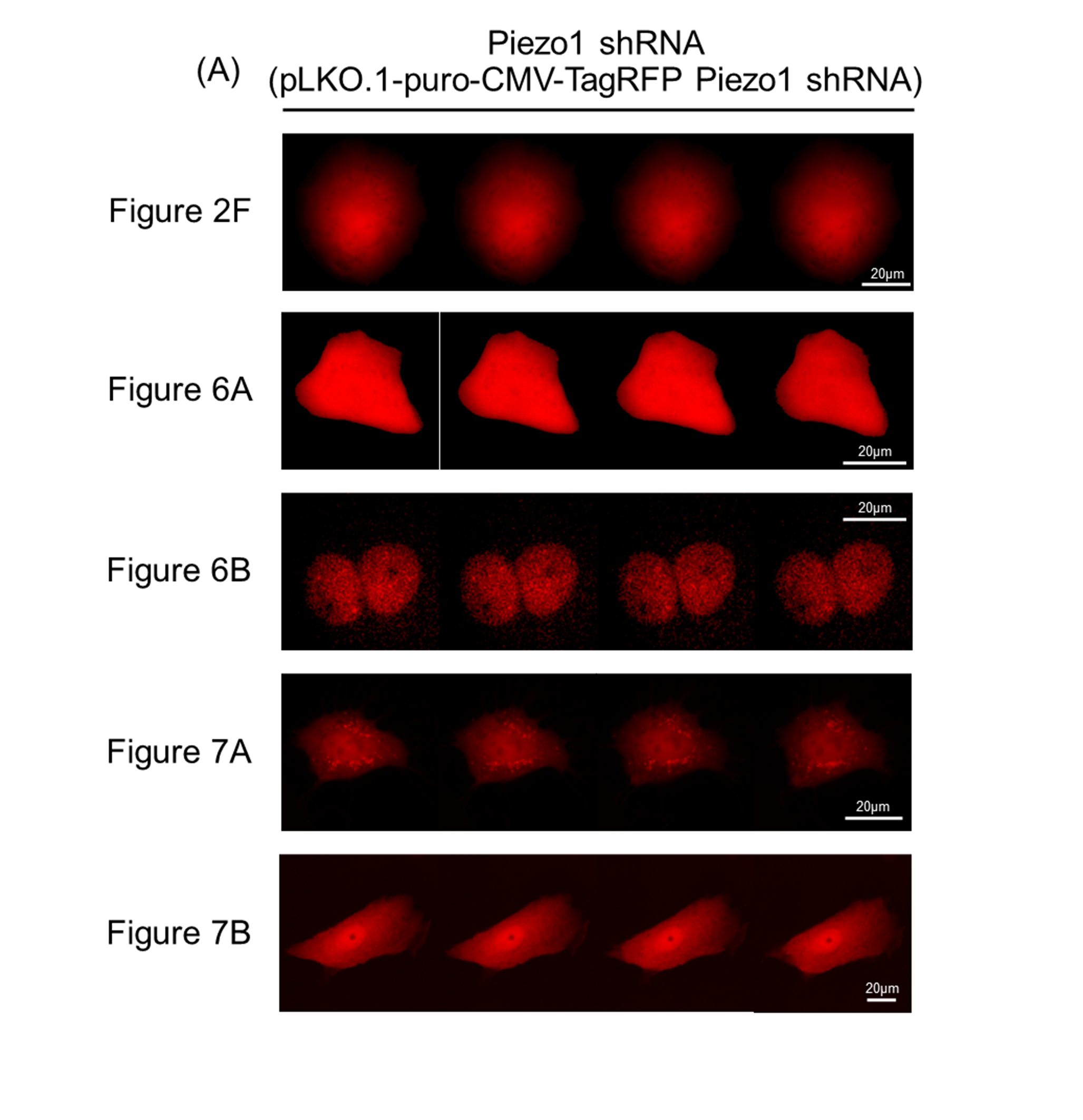

Supplement: Supplementary file 10 [file Image5.TIF]
