# Supplementary material for: Förster Resonance Energy Transfer-Based Single-Cell Imaging Reveals Piezo1-Induced Ca2+ Flux Mediates Membrane Ruffling and Cell Survival
Source: Front Cell Dev Biol. 2022 May 13;10:865056. doi: 10.3389/fcell.2022.865056 (PMC9136143; doi:10.3389/fcell.2022.865056)
Supplement: Supplementary file 14 [file DataSheet1.DOCX]

Supplementary Material

**Supplementary Figures**


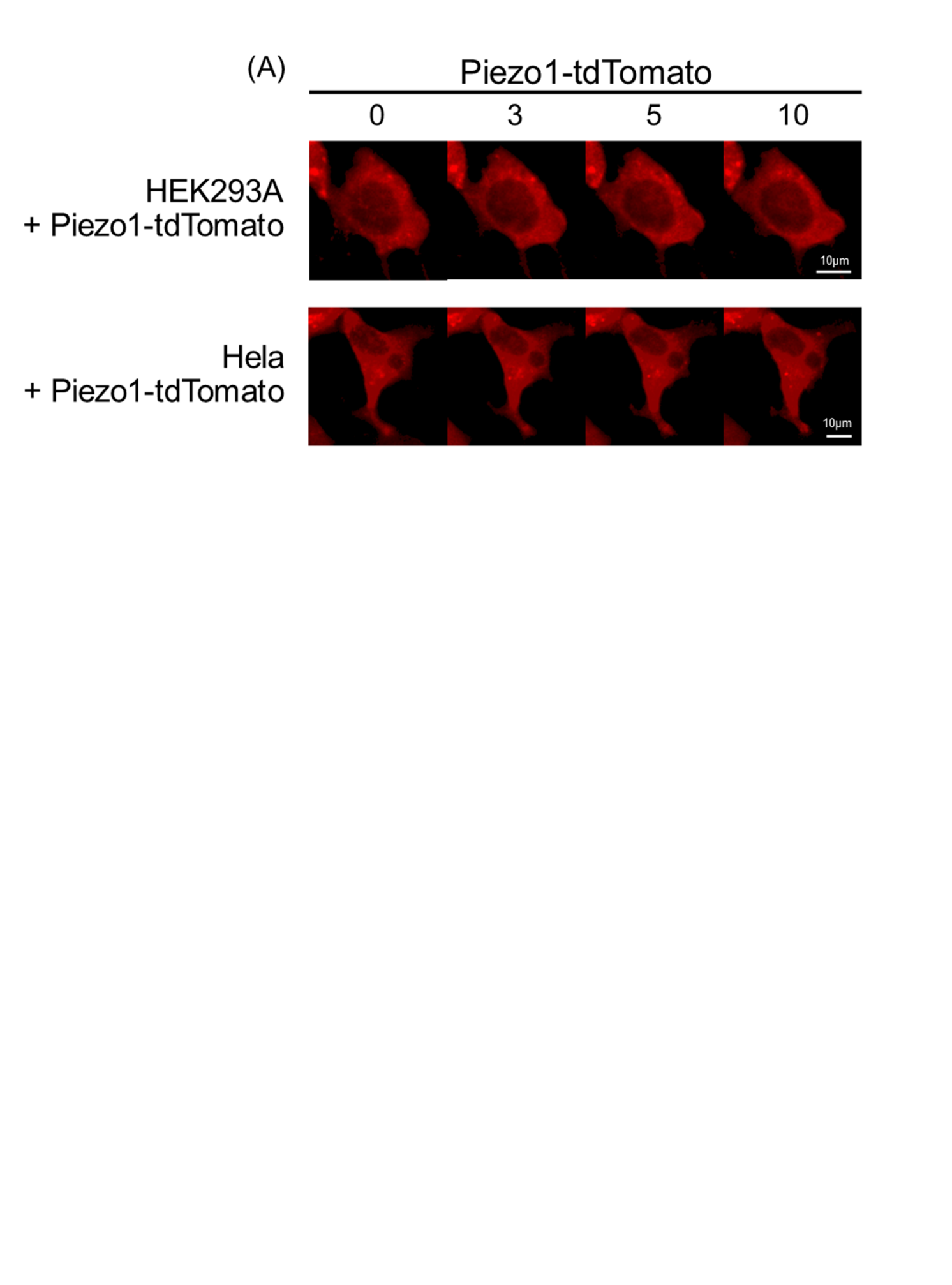


**Supplementary Figure S1.** **The red fluorescence images confirming the expression of Piezo1-tdTomato of cells in Figure 2h.** **(A)** Images of cells expressing Piezo1-tdTomato.


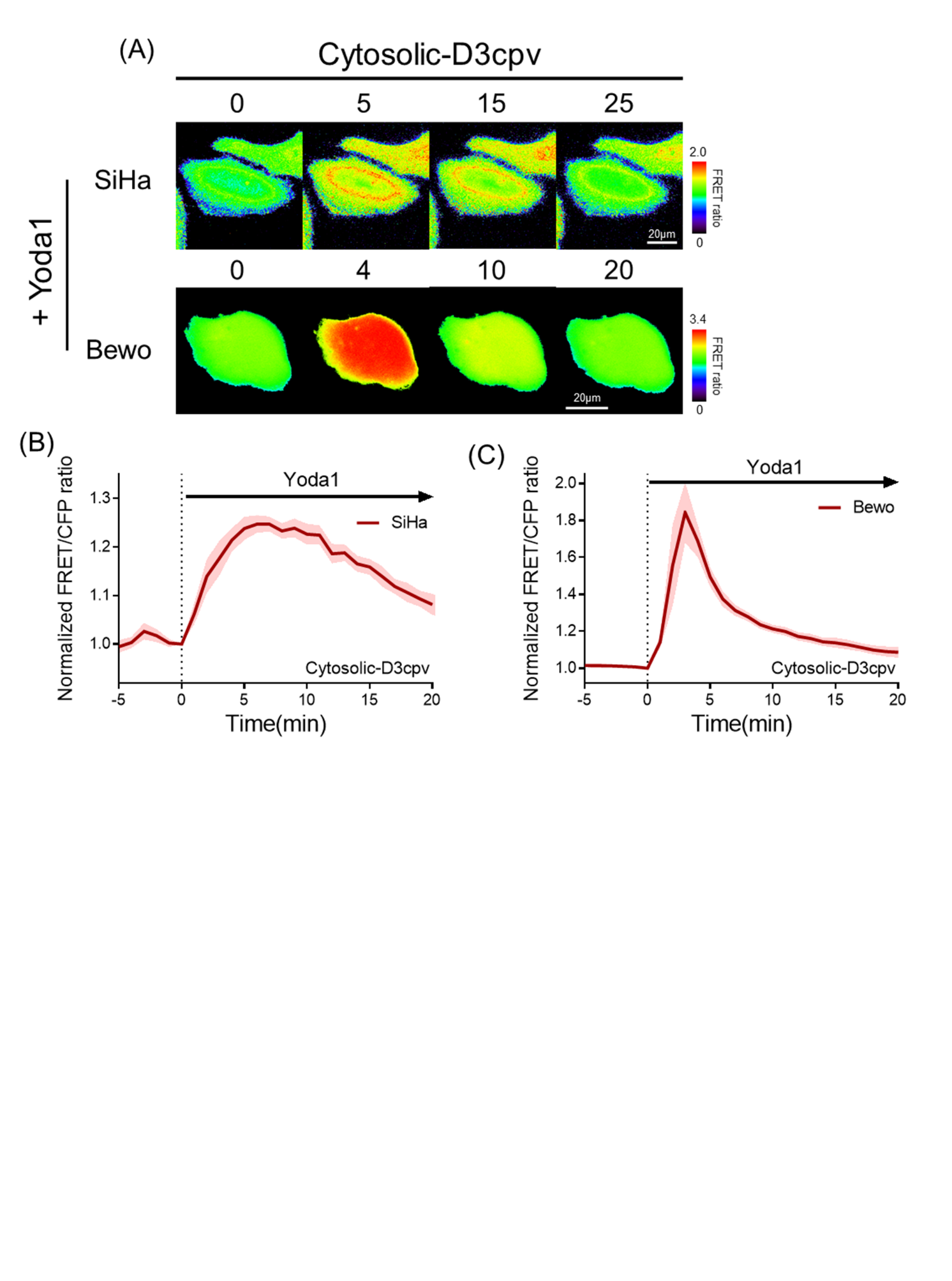


**Supplementary Figure S2.** **Yoda1-induced Ca^2+^ influx via Piezo1 in SiHa and BeWo cell lines.** **(A)** Time-lapse FRET images of the Cytosolic-D3cpv in SiHa and BeWo cells, which express Piezo1 endogenously. The cells were exposed to Yoda1 (1 μM) dissolved in CO_2_-independent culture medium. The color scale bars represent the range of the FRET/CFP emission ratio determined using the biosensor. Hot and cold colors indicate high and low Ca^2+^ concentrations, respectively. **(B, C)** The time courses represent the average of the normalized FRET/CFP emission ratio changes of cytosolic-D3cpv in **(B)** SiHa and **(C)** BeWo cells. The lines are mean values of normalized emission ratios, with diluted colors indicating the S.E.M (n=7).
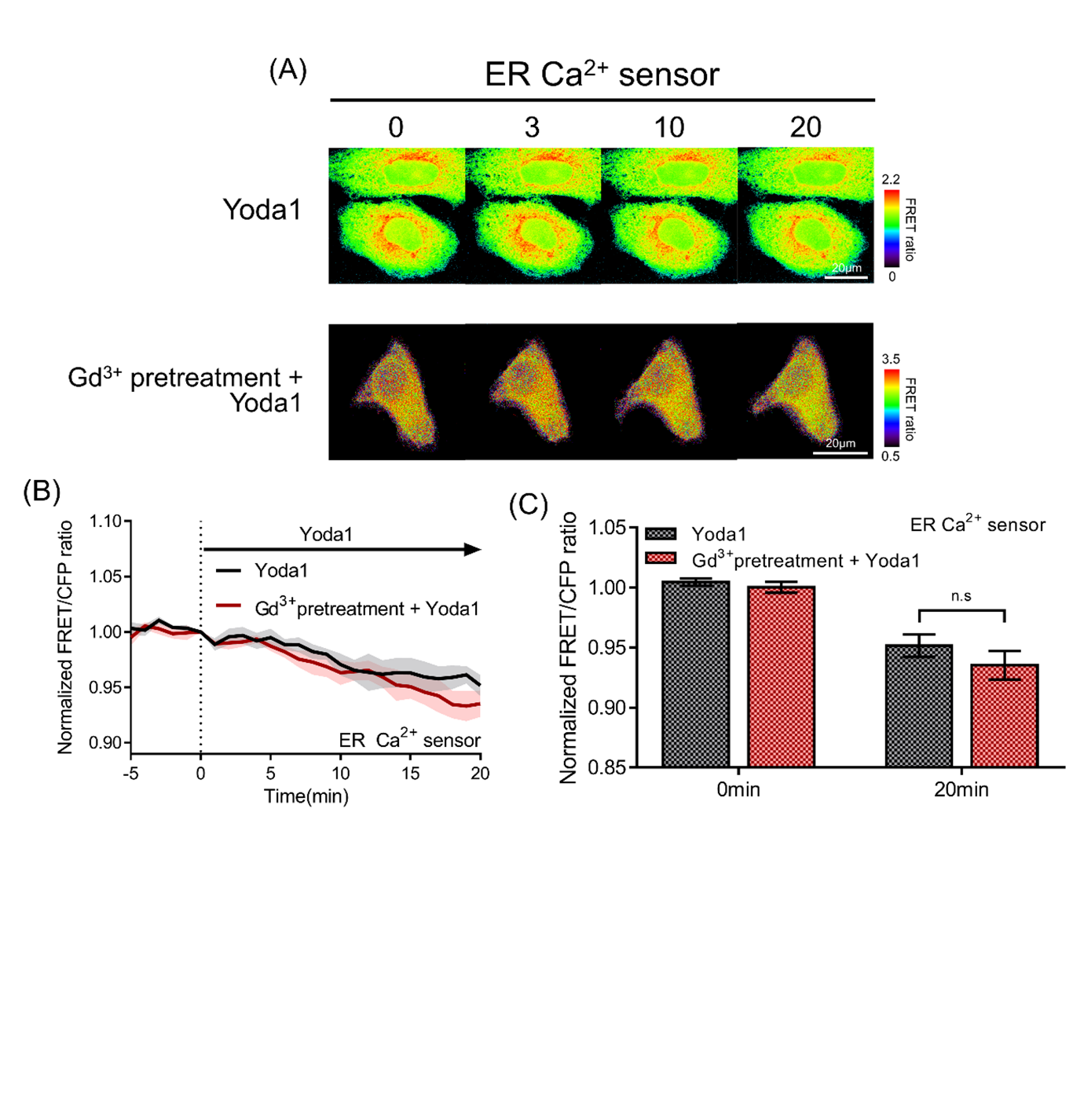


**Supplementary Figure S3. Gd^3+^ upregulates Piezo1-mediated ER-stored Ca^2+^ release by inhibiting extracellular calcium influx via Piezo1.** **(A)** Time-lapse FRET images of the ER Ca^2+^ sensor in untreated MCF-7 cells and cells pretreated with gadolinium (Gd^3+^). The cells were exposed to Yoda1 (1 μM) dissolved in a CO_2_-independent culture medium. The color scale bars represent the range of the FRET/CFP emission ratio determined using the biosensor. Hot and cold colors indicate high and low Ca^2+^ concentrations, respectively. **(B)** The time courses represent the average of the normalized FRET/CFP emission ratio changes of ER Ca^2+^ sensor. The lines are mean values of normalized emission ratios, with diluted colors indicating the S.E.M (n=7). **(C)** The bar graph describes the mean values of the normalized FRET/CFP emission ratios of the ER Ca^2+^ sensor at the described time with error bars indicating the S.E.M (n=7, n.s>0.05, Student's t-test).


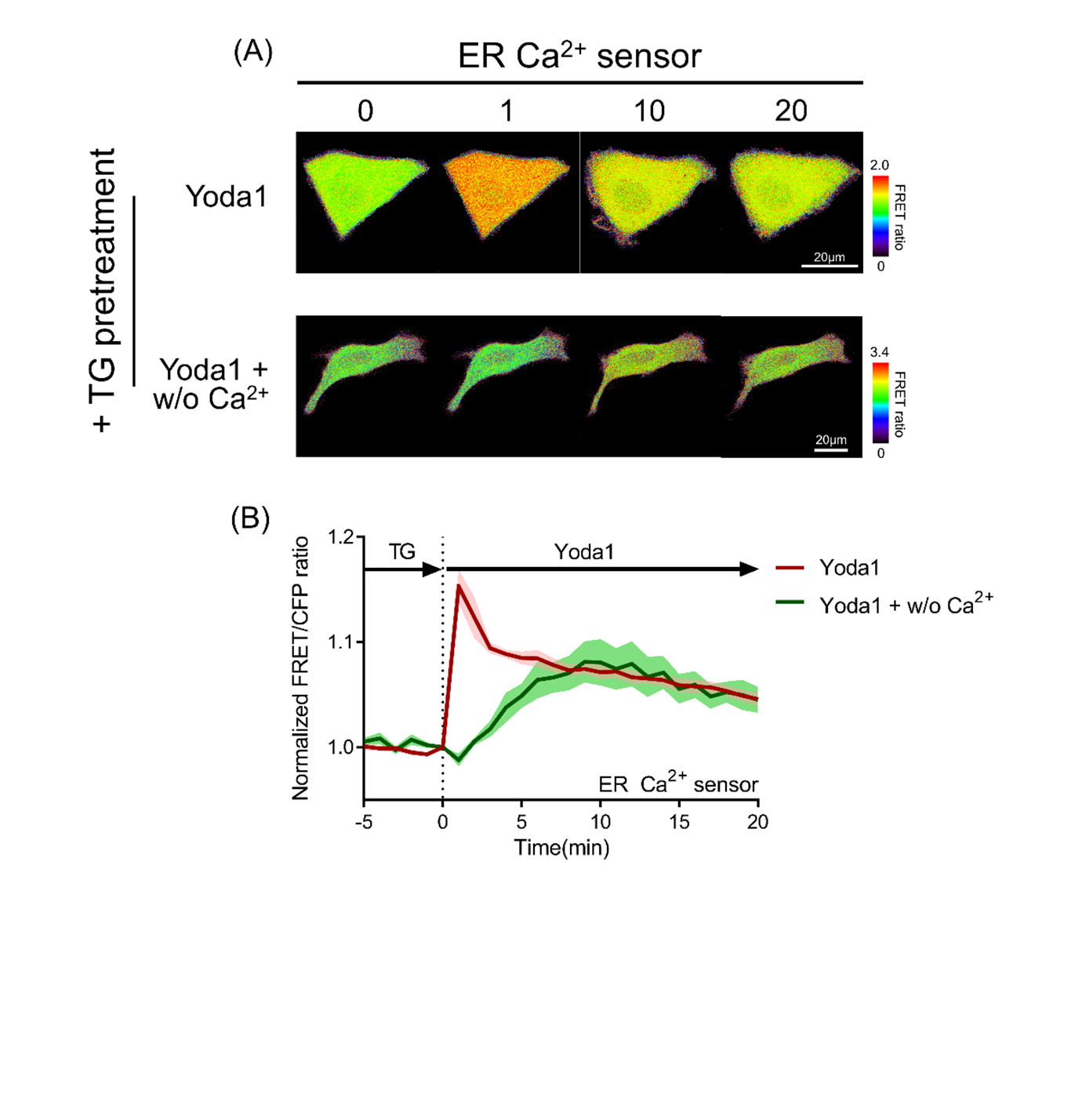


**Supplementary Figure S4. Yoda1 triggers Piezo1-mediated Ca^2+^ introduction into ER deficient in calcium.** **(A)** Time-lapse FRET images of the ER Ca^2+^ sensor in untreated MCF-7 cells and cells pretreated with thapsigargin (TG). The cells were exposed to Yoda1 (1 μM) dissolved in a CO_2_-independent culture medium. The color scale bars represent the range of the FRET/CFP emission ratio determined using the biosensor. Hot and cold colors indicate high and low Ca^2+^ concentrations, respectively. **(B)** The time courses represent the average of the normalized FRET/CFP emission ratio changes of ER Ca^2+^ sensor. The lines are mean values of normalized emission ratios, with diluted colors indicating the S.E.M (n=7).


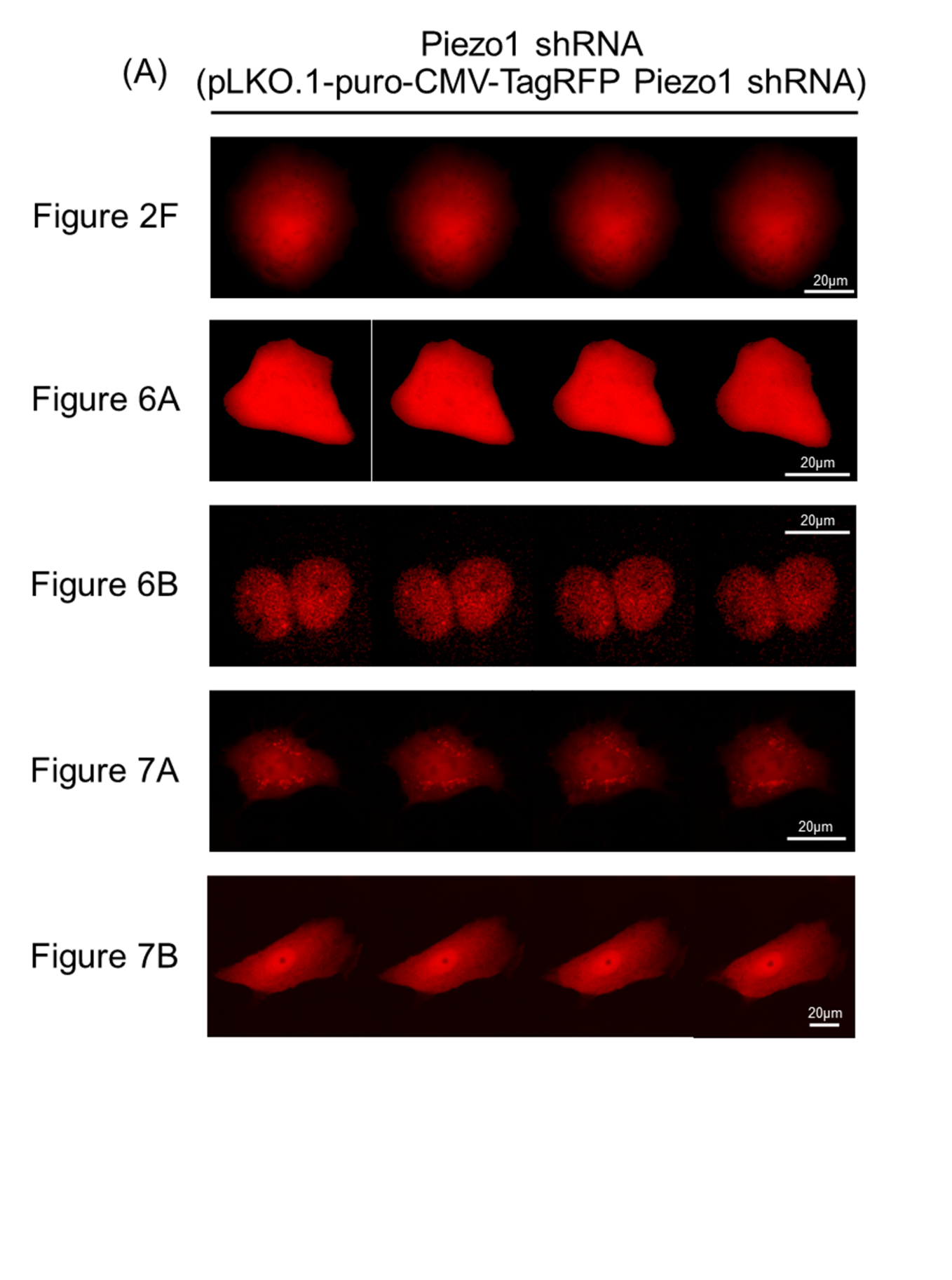


**Supplementary Figure S5.** **Red fluorescence images confirming the expression of Piezo1 shRNA in this study.** **(A)** Images of cells expressing Piezo1 shRNA which contains the TagRFP sequences.


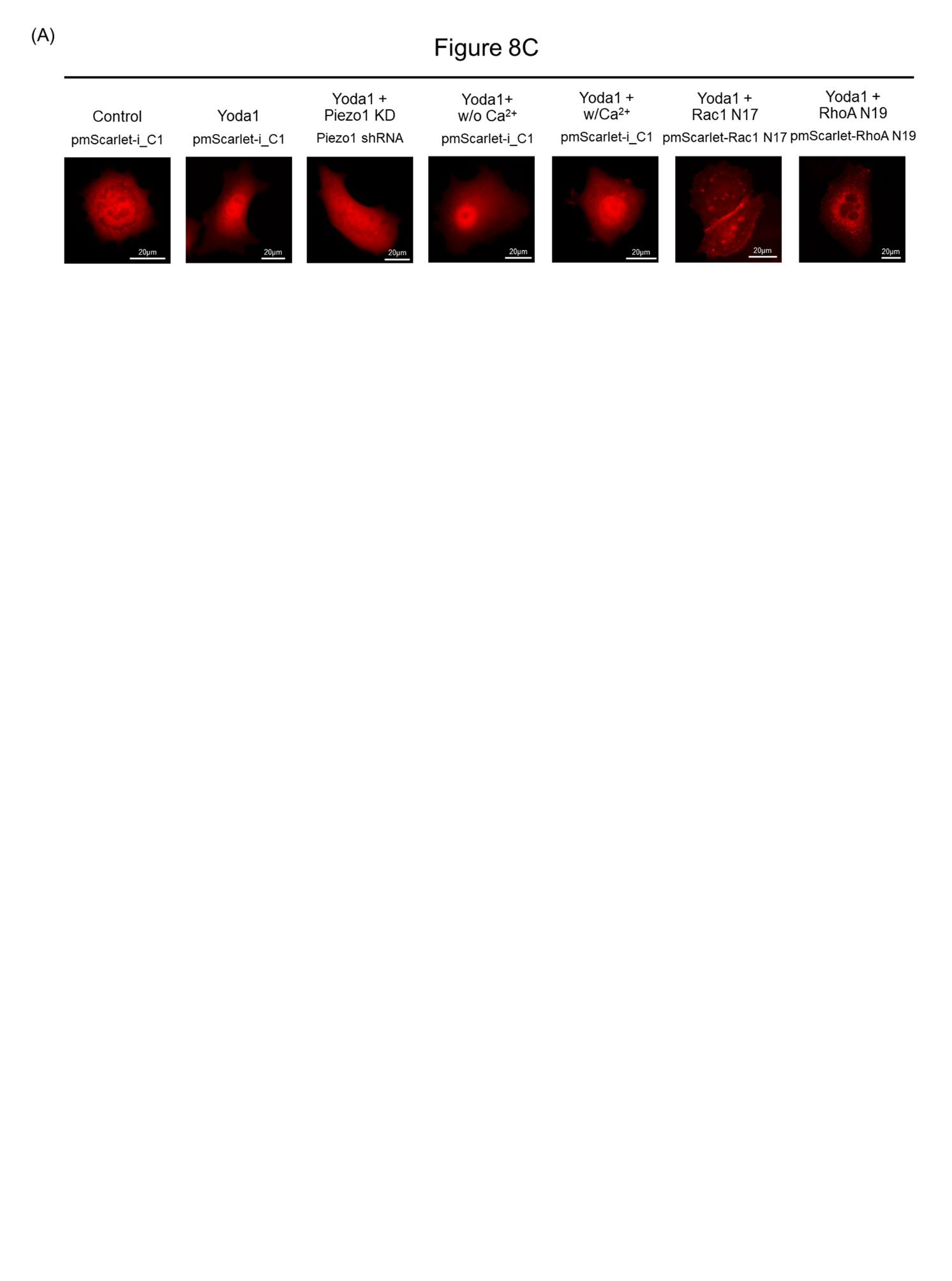


**Supplementary Figure S6.** **Red fluorescence images confirming the expression of introduced plasmid.** **(A)** Images of cells expressing each indicated plasmid which was tagged with red fluorescence protein. The pmScarlet-i_C1 was used as a control vector.

**Supplementary Tables**

**Supplementary Table S1. The detailed information of plasmids used in this study.**

| **Num** | **Plasmid name** | **Provided/Purchased by** | **Note** |
| --- | --- | --- | --- |
| **1** | Piezo1 shRNA (pLKO.1-puro-CMV-TagRFP Piezo1 shRNA) | Sigma | TRCN0000 121969 |
| **2** | Control shRNA (pRSI9-U6-(sh)-UbiC- TagRFP-2A-Puro) | Alex Chenchik & Gus Frangou | Addgene, #28289 |
| **3** | Piezo1-tdTomato | Dr. YingxiaoWang |  |
| **4** | Caveolin WT-EGFP | Dr. Jihye Seong  (KIST, Republic of Korea) |  |
| **5** | CAV1-P132L-mEGFP (Caveolin P132L-EGFP) | Ari Helenius | Addgene, #27708 |
| **6** | GFP-cortactin | Anna Huttenlocher | Addgene, #26722 |
| **7** | Cytosolic-D3cpv | Dr. YingxiaoWang (University of California, San Diego) |  |
| **8** | Lyn-D3cpv | Dr. Tae-Jin Kim |  |
| **9** | Kras-D3cpv | Dr. Tae-Jin Kim |  |
| **10** | ER Ca2+ sensor | Dr. Tae-Jin Kim |  |
| **11** | R-GECO1 | Robert Campbell | Addgene, #32444 |
| **12** | hyBRET-PKA-EV | Michiyuki Matsuda (Kyoto University) | Addgene, #108655 |
| **13** | Eevee-ROCK-Lyn | Michiyuki Matsuda |  |

**Supplementary Table S2. Detailed information on the primers used in this study.** All primers were manufactured and sequenced by Macrogen.

| **Num** | **Primer name** | **5’ – sequence – 3’** |
| --- | --- | --- |
| **1** | Piezo1 for | CAT CAT CCC CTT CAC GGC CC |
| **2** | Piezo1 rev | CCG GAG CTG CCC TCA ATC TG |
| **3** | GAPDH for | CGA CCA CTT TGT CAA GCT CA |
| **4** | GAPDH rev | AGG GGT CTA CAT GGC AAC TG |
| **5** | Rac1_XhoI_for | CTA ACT CGA GGT ATG CAG GCC ATC AAG |
| **6** | Rac1_EcoRI_rev | GGA CGA ATT CTT ACA ACA GCA GGC A |
| **7** | RhoA_XhoI_for | CTA ACT CGA GCT ATG GCT GCC ATC CGG |
| **8** | RhoA_EcoRI_rev | GGA CGA ATT CTC ACA AGA CAA GGC A |

**Supplementary Table S3. Detailed information and process of restriction enzyme cloning** All PCR products were directly sequenced for genetic confirmation by Macrogen.

| **Num** | **Used as** | **Plasmid name** | **Provided/ Purchased by** | **Used restriction enzymes** | **Note** |
| --- | --- | --- | --- | --- | --- |
| **1** | **EKAREV_NLS** | | | | |
|  | Vector | Eevee-ROCK-NLS | Michiyuki Matsuda | XhoⅠ,  NotⅠ | - |
|  | Insert | hyBRET-ERK-EV | Michiyuki Matsuda |  | Addgene, #108652 |
| **2** | **RaichuEV-Rac1-Rac CAAX** | | | | |
|  | Vector | RaichuEV-Rac1-Kras CAAX | Michiyuki Matsuda | XbaⅠ,  SalⅠ | - |
|  | Insert | Rac CAAX | Macrogen |  | Order by ourselves |
| **3** | **pmScarlet-Rac1 N17** | | | | |
|  | Vector | pmScarlet-i_C1 | Dr. YingxiaoWang | XhoⅠ, EcoRⅠ | - |
|  | Insert | Rac1 N17 | Dr. Jihye Seong |  | Conduct PCR with Primers (Num5, 6) |
| **4** | **pmScarlet-RhoA N19** | | | | |
|  | Vector | pmScarlet-i_C1 | Dr. YingxiaoWang | XhoⅠ, EcoRⅠ | - |
|  | Insert | RhoA N19 | Dr. Jihye Seong |  | Conduct PCR with Primers (Num7, 8) |

**Supplementary Table S4. The detailed filter sets of fluorescence channels used in this study.**

| **Used filter cube** | | | | |
| --- | --- | --- | --- | --- |
| **Num** | **Fluorescence Channel** | **Excitation**  **(nm)** | **Dichroic mirror**  **(nm)** | **Emission**  **(nm)** |
| CYR71010 filter cube | | | | |
| **1** | FRET | 436/28 | 459 | 539/24 |
| **2** | CFP | 436/28 | 459 | 473/22 |
| DFT51010 filter cube | | | | |
| **1** | TagRFP mScarlet tdTomato | 554/24 | 572 | 594/32 |
| **2** | EGFP | 479/33 | 500 | 519/25 |

**Supplementary Video S1-S7:**

**Supplementary Video S1.** The real-time membrane ruffling activity of Control group after Yoda1 treatment.

**Supplementary Video S2.** The real-time membrane ruffling activity of Yoda1 group after Yoda1 treatment.

**Supplementary Video S3.** The real-time membrane ruffling activity of Yoda1 + Piezo1 KD group after Yoda1 treatment.

**Supplementary Video S4.** The real-time membrane ruffling activity of Yoda1 + w/o Ca^2+^ group after Yoda1 treatment.

**Supplementary Video S5.** The real-time membrane ruffling activity of Yoda1 + w/ Ca^2+^ group after Yoda1 treatment.

**Supplementary Video S6.** The real-time membrane ruffling activity of Yoda1 + Rac1 N17 group after Yoda1 treatment.

**Supplementary Video S7.** The real-time membrane ruffling activity of Yoda1 + RhoA N19 group after Yoda1 treatment.
